# Exploring the properties of antituberculosis drugs through QSPR graph models and domination-based topological descriptors

**DOI:** 10.1038/s41598-024-73918-3

**Published:** 2024-10-17

**Authors:** Thilsath Parveen S, Balamurugan Bommahalli Jayaraman, Muhammad Kamran Siddiqui

**Affiliations:** 1 Department of Mathematics, School of Advanced Sciences, Vellore Institute of Technology, Chennai Campus, Chennai, Tamil Nadu 600127 India; 2 Department of Mathematics, COMSATS University Islamabad, Lahore Campus, Lahore, Pakistan

**Keywords:** Tuberculosis, QSPR analysis, Physicochemical properties, ADMET properties, Domination, Minimum domination distance, Domination distance-based topological indices, Biological techniques, Drug discovery, Molecular biology, Diseases, Health care, Chemistry, Mathematics and computing

## Abstract

Tuberculosis (TB) is a global health concern caused by the bacterium Mycobacterium tuberculosis. This infectious disease primarily affects the lungs but can also impact other organs. Effective TB control involves early diagnosis, appropriate treatment with a combination of antibiotics, and public health measures to prevent transmission. However, ongoing challenges include drug-resistant strains and socioeconomic factors influencing its prevalence. Drugs such as isoniazid, pyrazinamide, ethambutol, ethionamide, linezolid, and levofloxacin are approved for the treatment of drug-susceptible tuberculosis. The properties and other activities of the drug, can be analyzed by modelling its chemical structure in terms of a molecular graph $G=\left(V,E\right)$, by considering the atoms as the vertex set $V\left(G\right)$ and the bonds between the two atoms as the edge set $E\left(G\right)$. A molecular descriptor or topological index of $G$ represents the corresponding chemical molecule as a numerical value. Domination is one of the key concepts in the molecular structure used to analyze the properties of atoms. In this article, the domination distance-based topological indices of the drugs isoniazid, pyrazinamide, ethambutol, ethionamide, linezolid, and levofloxacin are computed to conduct QSPR (Quantitative Structure–Property Relationship) analysis, exploring their physicochemical and ADMET (Absorption, Distribution, Metabolism, Excretion, and Toxicity) properties. Quadratic regression is then used in the QSPR analysis to examine the physicochemical and ADMET properties of these drugs. The results of this analysis indicate that the domination Schultz index and domination SM index are the indices most strongly correlated with the majority of the physicochemical and ADMET properties. The QSPR analysis can also be extended to analogs of these drugs and to other treatment drugs, such as rifampin and rifapentine, to further explore their properties.

## Introduction

Tuberculosis (TB) is a deadly disease caused by Mycobacterium tuberculosis and it can be cured with right antibiotic. TB is a contagious bacterial infection. It primarily affects the lungs but can also target other parts of the body. TB is primarily transmitted through air, when an infected person coughs or sneezes. The key symptoms of TB are persistent cough, chest pain, coughing with blood, fatigue, fever, night sweats, and unintentional weight loss. TB disease if not treated properly turns out to be fatal. The two TB related disorders are active TB(ATB) and latent TB (LTB). Active TB often referred to as TB disease, is contagious and its symptoms differ depending on whether it affects the lungs or extrapulmonary organs. Active TB poses a life-threatening risk if untreated. It is symptomatic and contagious. In contrast, latent TB refers to the presence of inactive TB bacteria within the body. Latent tuberculosis is asymptomatic and not contagious. Individuals with weakened immune systems, often due to medication, are at a higher risk of developing latent TB. Various methods of detecting and diagnosing TB and managing this disease effectively^1^. However, laboratory studies have been conducted to investigate the potential antibacterial effects of drugs such as isoniazid, pyrazinamide, ethambutol, ethionamide, linezolid and levofloxacin.

Patients with ATB are given isoniazid^2^, which is used to treat or prevent the reactivation of infectious TB. It functions by preventing the TB bacteria from reproducing and spreading. Pyrazinamide^3^ is a drug given to TB patients to slow down the growth of certain bacteria causing TB. Ethambutol^4^ is a drug used to treat pulmonary tuberculosis and as a medication that inhibits the growth of bacteria. It interferes the way in which bacteria build their cell wall and this interaction stops the bacteria from growing properly. Ethambutol and ethionamide are drugs that should not be used alone and should be combined with any other antituberculosis medicine. Ethionamide is an antibiotic that fights against bacteria and is used to treat multidrug-resistant tuberculosis. Linezolid^5^ is an effective multidrug-resistant antibiotic that can be used to optimize infection-control methods and prevent their spread. Levofloxacin, a fluoroquinolone antibacterial drug, suggested over all other fluoroquinolones and used in the second-line antitubercular treatment because it has more in-vitro action against Mycobacterium tuberculosis^6^.

Chemical graph theory^7^ bridges the gap between chemistry and mathematics, enabling scientists to compute relationships between molecular structure, properties, and reactivity through graph-based models and computational techniques. The chemical molecules and their structures are represented as a molecular graph or chemical graph, $G\left(V, E\right)$ with atoms as the vertex set $V\left(G\right)$ and the bonds between the atoms as the edge set $E\left(G\right)$. In this modelling, double bonds are considered as multiple edges between the atoms. The degree of the vertex (atom) $v$, denoted by $d\left(v\right)$, is the number of edges (bonds) incident to the vertex $v$. The distance between the vertices $u$ and $v$,$d\left(u,v\right)$ is the number of edges in the shortest path between them.

Topological descriptors often referred as molecular descriptors^8^, assist in the transformation of structural information of chemical molecules into quantitative form. Topological indices provide a numerical value based on their graph structure. From these indices, it is possible to analyze and investigate some physicochemical properties and ADMET properties of a molecule. Different topological indices have been defined based on various graph structural parameters. They comprise distance-based indices, degree-based indices, connectivity-based indices, neighborhood-based indices, eigenvalue-based indices etc. Topological indices are used in analyzing the Quantitative Structure-Property Relationship (QSPR) and Quantitative Structure-Activity Relationship (QSAR) for various chemical compounds have been studied in^9–12^. Furthermore, the QSPR graph domination model with various domination numbers is introduced and explored for anti-fungal drugs^13^. Various topological coindices, computed using a polynomial approach for various diseases, are studied in^14–19^.

Motivated by research on various distance-based topological indices, this article explores several domination distance-based topological indices. These indices are studied using the concept of domination in graphs, where a subset of atoms dominates the chemical graph. Domination-based indices offer unique insights into the structural characteristics and connectivity patterns of chemical graphs.Domination in graphs is an important concept of graph theory, due to its significance in molecular structure, decision-making, and its interconnectedness with various other graph concepts. Berge^20^ and Ore^21^ initiated the concept of domination and contributed significantly to the development and understanding the concepts of domination in graphs. In the chemical graph $G\left(V,E\right)$, a subset S of the atom set $V$ is called as a Dominating Set (DS) if, for any atom $v \in \left(V- S\right),$ there exists an atom $u \in S$ such that $u$ and $v$ are neighbouring atoms. More models related to domination in graphs, researchers can refer to^22–28^. The minimum dominating set^29^ is the dominating set of smallest size. Domination finds wide-ranging applications across diverse domains, including the physical, biological, and social sciences, as well as discrete optimization and classical algebraic problem-solving for real-world challenges. Furthermore, the concept of domination topological index in graphs was introduced by Hanan et al. in^30^. Farhani introduced Schultz polynomial^31^ in benzene molecules. In 2019, Jayalalitha et al. introduced Schultz index and modified Schultz index using minimum dominating distance matrix. Various dominating distance-based indices such as domination Weiner index^32^, domination hyper Weiner index^33^, domination Schultz index^34^, domination modified Schultz index^34^, have been studied in the literature using minimum dominating distance matrix. The domination distance indices such as domination Harary index, domination terminal Weiner index, domination Ashwini index and domination SM index of molecular graphs are introduced in this article.

## Domination in chemical graphs

In this section, the concepts and definitions related to domination in chemical graphs are considered. Let $G=\left(V,E\right)$ be a chemical graph with vertex set $V$ (set of atoms) and edge set $E$(set of bonds).

### Definition 2.1

Haynes et al.^27^ Let $\text{G}\left(\text{V},\text{ E}\right)$ be a molecular graph of order n with atom set $\text{V}$ and bond set $\text{E}$. A dominating set is a subset $S$ of $V$ such that every vertex in $V-S$ is adjacent to an element in $\text{S}$ and two vertices in $\text{S}$ are adjacent. The domination number of $G$, denoted by $\gamma \left(\text{G}\right)$, is the minimum size of a dominating set in $\text{G}$.

### Definition 2.2

Vijayalakshmi et al.^35^ Let $\text{G}$ be a molecular graph of order $\text{n}$ with atom set $\text{V}$ and bond set $\text{E}$. A dominating set is a subset $S$ of $V$ such that every vertex in $V-S$ is adjacent to an element in $\text{S}$. The minimum dominating distance matrix of the graph $\text{G}$ is a $n\times n$ matrix, where

$$D{d}_{ij}\left(G\right)=\left\{\begin{array}{cc}\text{min}\left(d\left({v}_{i}, {v}_{j}\right)\right)& if \ \ i\ne j\\ 1& if \ i=j \ and\ {v}_{i}\in S.\end{array}\right.$$

### Example 2.1

**Fig. 1:**
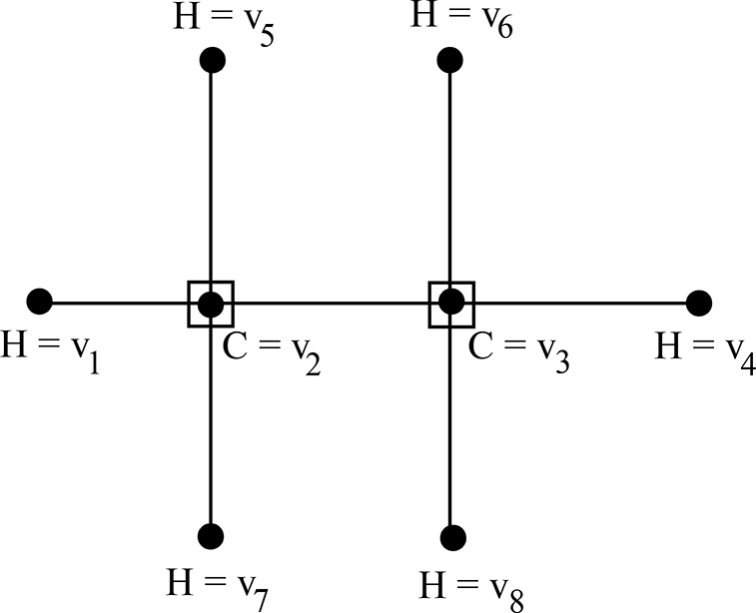
Molecular Graph of Ethane.

The molecular graph of ethane ${C}_{2}{H}_{6},$ with atom set $V=\left\{{v}_{1}, {v}_{2},\dots ,{v}_{8}\right\}$ is shown in Fig. 1. The minimum dominating set of ethane is $S=\left\{{v}_{2},{v}_{3}\right\},$ and therefore its domination number is 2.

The minimum dominating distance matrix of the ethane is

$$D{d}_{ij}\left({C}_{2}{H}_{6}\right)=\left(\begin{array}{cccccccc}0& 1& 2& 3& 2& 3& 2& 3\\ 1& 1& 1& 2& 1& 2& 1& 2\\ 2& 1& 1& 1& 2& 1& 2& 1\\ 3& 2& 1& 0& 3& 2& 3& 2\\ 2& 1& 2& 3& 0& 3& 2& 3\\ 3& 2& 1& 2& 3& 0& 3& 2\\ 2& 1& 2& 3& 2& 3& 0& 3\\ 3& 2& 1& 2& 3& 2& 3& 0\end{array}\right)$$

## Domination distance—based topological indices of molecular graphs

Let $\text{G}\left(\text{V},\text{ E}\right)$ be a molecular graph where $V$ is the set of atoms and $E$ is the set of bonds between atoms. The various domination distance-based topological indices for a molecular graph $G$ obtained through dominating polynomial and dominating distance-based matrix are defined in this section.

The dominating Schultz polynomial and domination modified Schultz polynomial^34^ are given by

1 $$DSC\left(G\right)=\frac{1}{2}\sum_{{v}_{i}{ ,v}_{j}\in V\left(G\right)}[{(deg}_{D}{(v}_{i}){+ deg}_{D}{(v}_{j})] {x}^{{d}_{D}\left({v}_{i} , {v}_{j}\right)}$$

2 $$DS{C}^{*}\left(G\right)=\frac{1}{2}\sum_{{v}_{i},{v}_{j}\in V\left(G\right)}[{(deg}_{D}{(v}_{i}){\times deg}_{D}{(v}_{j})] {x}^{{d}_{D}\left({v}_{i} , {v}_{j}\right)}$$

The domination Schultz index and domination modified Schultz index are

$$DSC\left(G\right)=$$ $$\frac{\partial \left(DSC\left(G, x\right)\right)}{\partial x}|x=1$$ and $$DS{C}^{*}\left(G\right)=\frac{\partial \left(DS{C}^{*}\left(G, x\right)\right)}{\partial x}|x=1$$.

The domination distance-based topological descriptors viz., Weiner index^32^ and hyper Weiner index^33^ are obtained using minimum dominating distance - based matrix. Domination Harary index, domination terminal Weiner index, domination Ashwini index and domination SM index of molecular graphs are introduced in this article.

(i) Domination Weiner index of $G$ denoted by $DW\left(G\right),$ is defined as
  $$DW\left(G\right)=\frac{1}{2}\sum_{{v}_{i, }{v}_{j} \in V\left(G\right)}{d}_{D}{(v}_{i}, {v}_{j})$$
(ii) Domination hyper Weiner index of $G$ denoted by $DHW\left(G\right)$, is defined as
  $$DHW\left(G\right)=\frac{1}{2}\sum_{{v}_{i}, {v}_{j}\in V\left(G\right) }\left[{{d}_{D}{(v}_{i}, {v}_{j})+d}_{D}{\left({v}_{i}, {v}_{j}\right)}^{2}\right]$$
(iii) Domination terminal Weiner index of $G$ denoted by $DTW\left(G\right)$, is defined as
  $$DTW\left(G\right)=\frac{1}{2}\sum_{{v}_{i, }{v}_{j} \in {V}_{T}\left(G\right)}{d}_{D}{(v}_{i}, {v}_{j})$$
  where *T* denotes the set of all pendant vertices of the graph $G$.
(iv) Domination Harary index of $G$ denoted by $DH\left(G\right),$ is defined as
  $$DH\left(G\right)=\frac{1}{2}\sum_{{v}_{i, }{v}_{j} \in V\left(G\right)}\frac{1}{{d}_{D}{(v}_{i}, {v}_{j})}$$
(v) Domination Ashwini index of $G$ denoted by $DA\left(G\right),$ is defined as
  $$DA\left(G\right)=\sum_{1\le i<j\le n}{d}_{T}\left( {v}_{i}, {v}_{j}\right)\left[{\text{deg}}_{T}\left(N\left({v}_{i}\right)\right)+{\text{deg}}_{T}\left(N\left({v}_{j}\right)\right)\right]$$
(vi) Domination SM index of $G$ denoted by $DSM\left(G\right),$ is defined as
  $$DSM\left(G\right)=\sum_{1\le i<j\le n}{d}_{T}\left( {v}_{i}, {v}_{j}\right)\left[{\text{deg}}_{T}\left(N\left({v}_{i}\right)\right)\times {\text{deg}}_{T}\left(N\left({v}_{j}\right)\right)\right]$$

These indices are used to carry out QSPR (Quantitative Structure Property Relationship) analysis to compute the physicochemical and ADMET properties of the drugs used to treat the disease.

## Computation of domination numbers for chemical graphs

In this section, domination numbers and domination indices of chemical graphs such as isoniazid, pyrazinamide, ethambutol, ethionamide, linezolid and levofloxacin were computed. The chemical structures of these drugs are collected from the website https://pubchem.ncbi.nlm.nih.gov/.The chemical graphs were obtained from the chemical structures of each of the above drugs. The domination distance-based indices are computed through minimum dominating distance-based matrix calculated for each of the drugs.

### Theorem 4.1

*Let* $${G}_{1}$$ *be the chemical graph of the drug isoniazid. The domination distance based topological indices of the graph*$${G}_{1}$$*are*$$DW\left({G}_{1}\right)=252.5;$$$$DHW\left({G}_{1}\right)=1261;$$$$DH\left({G}_{1}\right)=36.20238;$$$$DSC\left({G}_{1}\right)= 1271;$$$$DS{C}^{*}\left({G}_{1}\right)=1537;$$$$DTW\left({G}_{1}\right)=8;$$$$DA\left({G}_{1}\right)=48;$$$$DSM\left({G}_{1}\right)=72.$$

### Proof

**Fig. 2:**
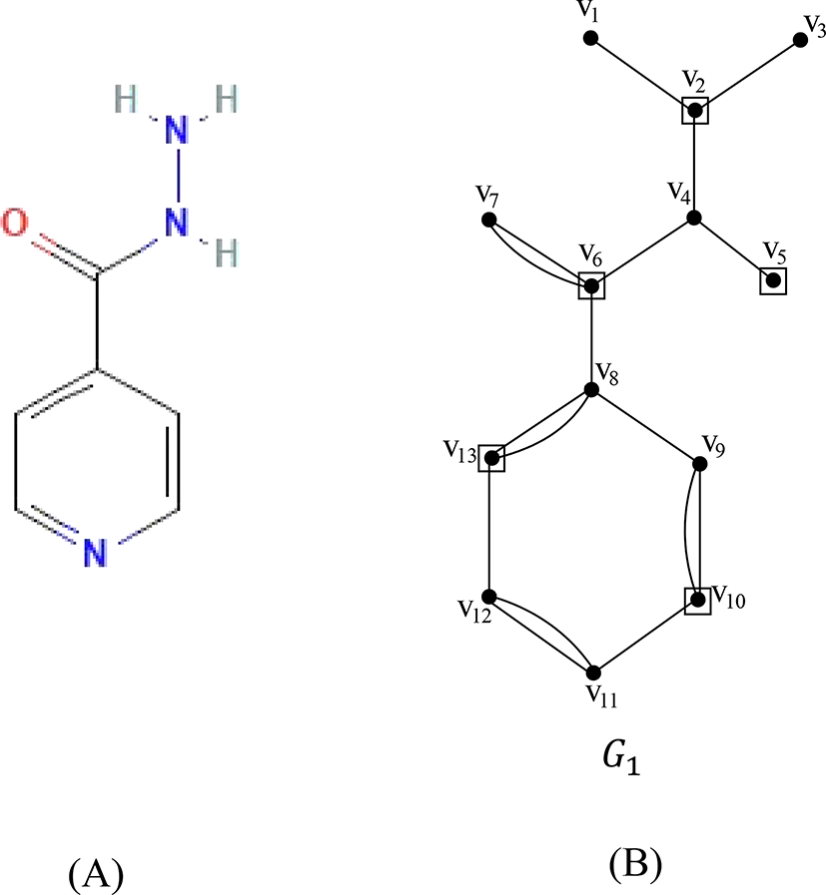
(**A**) Molecular structure of isoniazid (**B**) The Chemical graph of isoniazid.

Let ${G}_{1}\left({V}_{1},{E}_{1}\right)$ be the chemical graph of the drug isoniazid with 13 atoms(vertices) and 17 bonds (edges) between the atoms. The chemical structure of isoniazid and its corresponding chemical graph ${G}_{1}$ are shown in Fig. 2(A) and (B) respectively.

Let ${V}_{1}=\left\{{v}_{1}, {v}_{2}, {v}_{3},\dots , {v}_{12},{v}_{13}\right\}$ be the atom set of ${G}_{1}$. Let the partition of ${V}_{1}$ be.

${C}_{1}=\left\{{v}_{1}, {v}_{2}, {v}_{3}, {v}_{4}, {v}_{5},{v}_{6}, {v}_{7}\right\}$ and ${C}_{2}=\left\{{v}_{8}, {v}_{9}, {v}_{10}, {v}_{11}, {v}_{12}, {v}_{13}\right\}$ where ${C}_{1}\cup {C}_{2}={V}_{1}{, C}_{1}\cap {C}_{2}=\varnothing$. The set ${C}_{1}$ contains a set of pendant atoms and adjacent atoms linked with the pendant atoms and ${C}_{2}$ contains atoms in the benzene ring together with cut edges/bonds of ${G}_{1}$. In the set ${C}_{1}$ the vertices with maximum degree such as $\left\{{v}_{2},{v}_{5}, {v}_{6}\right\}$ constitutes a dominating set and in the set ${C}_{2}$ any two non-adjacent atoms in the benzene ring such as $\left\{{v}_{10}, {v}_{13}\right\}$ forms a dominating set. The dominating set of ${C}_{1}$ and ${C}_{2}$ are added to get the dominating set of the graph ${G}_{1}$. The dominating set of the graph ${G}_{1}$ is $\left\{{v}_{2},{v}_{5}, {v}_{6}, {v}_{10}, {v}_{13}\right\}$ and therefore the domination number of ${G}_{1}$ is $\gamma \left({G}_{1}\right)= 5$.

The minimum dominating distance matrix of the molecular graph ${G}_{1}$ is obtained using the dominating set of ${G}_{1}$.The minimum dominating distance matrix of ${G}_{1}$ is $D{d}_{ij}\left({G}_{1}\right)$ and is given by

$$D{d}_{ij}\left({G}_{1}\right)=\left(\begin{array}{cccccccccccccc}V& {v}_{1}& {v}_{2}& {v}_{3}& {v}_{4}& {v}_{5}& {v}_{6}& {v}_{7}& {v}_{8}& {v}_{9}& {v}_{10}& {v}_{11}& {v}_{12}& {v}_{13}\\ {v}_{1}& 0& 1& 2& 2& 3& 3& 4& 4& 5& 6& 7& 6& 5\\ {v}_{2}& 1& 1& 1& 1& 2& 2& 3& 3& 4& 5& 6& 5& 4\\ {v}_{3}& 2& 1& 0& 2& 3& 3& 4& 4& 5& 6& 7& 6& 5\\ {v}_{4}& 2& 1& 2& 0& 1& 1& 2& 2& 3& 4& 5& 4& 3\\ {v}_{5}& 3& 2& 3& 1& 1& 2& 3& 3& 4& 5& 6& 5& 4\\ {v}_{6}& 3& 2& 3& 1& 2& 1& 1& 1& 2& 3& 4& 3& 2\\ {v}_{7}& 4& 3& 4& 2& 3& 1& 0& 2& 3& 4& 5& 4& 3\\ {v}_{8}& 4& 3& 4& 2& 3& 1& 2& 0& 1& 2& 3& 2& 1\\ {v}_{9}& 5& 4& 5& 3& 4& 2& 3& 1& 0& 1& 2& 3& 2\\ {v}_{10}& 6& 5& 6& 4& 5& 3& 4& 2& 1& 1& 1& 2& 3\\ {v}_{11}& 7& 6& 7& 5& 6& 4& 5& 3& 2& 1& 0& 1& 2\\ {v}_{12}& 6& 5& 6& 4& 5& 3& 4& 2& 3& 2& 1& 0& 1\\ {v}_{13}& 5& 4& 5& 3& 4& 2& 3& 1& 2& 3& 2& 1& 1\end{array}\right)$$

The degree of vertices of the molecular graph $${G}_{1}$$ are $$\text{deg}\left({v}_{1}\right)=1;$$$$\text{deg}\left({v}_{2}\right)=3;$$$$\text{deg}\left({v}_{3}\right)=1;$$$$\text{deg}\left({v}_{4}\right)=3;$$$$\text{deg}\left({v}_{5}\right)=1;$$$$\text{deg}\left({v}_{6}\right)=4;$$$$\text{deg}\left({v}_{7}\right)=2;$$$$\text{deg}\left({v}_{8}\right)=4;$$$$\text{deg}\left({v}_{9}\right)=3;$$$$\text{deg}\left({v}_{10}\right)=3;$$$$\text{deg}\left({v}_{11}\right)=3;$$$$\text{deg}\left({v}_{12}\right)=3;$$$$\text{deg}\left({v}_{13}\right)=3.$$

For every pair of vertices ${v}_{i}, {v}_{j}$ of the vertex set ${V}_{1}$, the summation values of $\left(d{v}_{i}+d{v}_{j}\right)$ and ($d{v}_{i}\times d{v}_{j})$ of isoniazid are computed from the minimum dominating distance matrix and degree of vertices and are summarized in Table 1.

**Table 1:** The values $\sum_{{v}_{i}, {v}_{j} \in {G}_{1}}\left(d{v}_{i}+d{v}_{j}\right)$ and $\sum_{{v}_{i}, {v}_{j} \in {G}_{1}}\left(d{v}_{i}\times d{v}_{j}\right)$ with different distances of the molecular graph ${G}_{1}$ of isoniazid.

The distance $d\left({v}_{i}, {v}_{j}\right)$	1	2	3	4	5	6	7
$\sum_{{v}_{i}, {v}_{j} \in {G}_{1}}\left(d{v}_{i}+d{v}_{j}\right)$	210	192	178	260	235	52	16
$\sum_{{v}_{i}, {v}_{j} \in {G}_{1}}\left(d{v}_{i}\times d{v}_{j}\right)$	316	272	236	156	102	48	12

By using the values in Table 1, the domination Schultz polynomial and domination modified Schultz polynomial of the drug isoniazid are given by

The domination Schultz polynomial is given is

$$DSC\left({G}_{1}, x\right)=\frac{1}{2}\sum_{{v}_{i}{v}_{j}\in V\left({G}_{1}\right)}[{(deg}_{D}{(v}_{i}){+ deg}_{D}{(v}_{j})] {x}^{{d}_{D}\left({v}_{i} , {v}_{j}\right)}$$

3 $$DSC\left({G}_{1}, x\right)=105x+96{x}^{2}+89{x}^{3}+65{x}^{4}+47{x}^{5}+26{x}^{6}+8{x}^{7}$$

The domination modified Schultz polynomial is given is

$$DS{C}^{*}\left({G}_{1}, x\right)=\frac{1}{2}\sum_{{v}_{i}{v}_{j}\in V\left({G}_{1}\right)}[{(deg}_{D}{(v}_{i}){\times deg}_{D}{(v}_{j})] {x}^{{d}_{D}\left({v}_{i} , {v}_{j}\right)}$$

4 $$DS{C}^{*}\left({G}_{1},x\right)=158x+136{x}^{2}+118 {x}^{3}+78 {x}^{4}+51 {x}^{5}+24 {x}^{6}+6{x}^{7}$$

The domination Schultz index and domination modified Schultz index are obtained from Eqs. (3) and (4) respectively.

The domination Schultz index $$\left(DSC\right)$$ is $$\left[{\left.\frac{\partial \left(DSC\left({G}_{1}, x\right)\right)}{\partial x}\right|}_{x=1}\right]$$ and therefore $$\frac{\partial \left(DSC\left({G}_{1}, x\right)\right)}{\partial x}=$$$$105+192x+267{x}^{2}+260{x}^{3}+235{x}^{4}+156{x}^{5}+56 {x}^{6}$$ and hence $$\left[{\left.\frac{\partial \left(DSC\left({G}_{1}, x\right)\right)}{\partial x}\right|}_{x=1}\right]=1271$$.

The domination modified Schultz index $$\left(DS{C}^{*}\right)$$ is $$\left[{\left.\frac{\partial \left(DS{C}^{*}\left({G}_{1}, x\right)\right)}{\partial x}\right|}_{x=1}\right]$$ and therefore $$\frac{\partial \left(DS{C}^{*}\left({G}_{1}, x\right)\right)}{\partial x}=$$$$158+272x+354{x}^{2}+312{x}^{3}+255{x}^{4}+144{x}^{5}+42{x}^{6}$$ and hence $$\left[{\left.\frac{\partial \left(DS{C}^{*}\left({G}_{1}, x\right)\right)}{\partial x}\right|}_{x=1}\right]=1537$$.

The domination distance based topological indices are computed using minimum dominating distance matrix, $D{d}_{ij}\left({G}_{1}\right)$ for the drug isoniazid and are given as follows:

(i) The domination Weiner index of the molecular graph ${G}_{1}$ is computed as
  $$DW\left({G}_{1}\right)=\frac{1}{2}\sum_{{v}_{i, }{v}_{j} \in V\left({G}_{1}\right)}{d}_{D}{(v}_{i}, {v}_{j})=\frac{1}{2}\left[505\right]=252.5$$
(ii) The domination hyper Weiner index of the molecular graph ${G}_{1}$ is calculated as
  $$DHW\left({G}_{1}\right)=\frac{1}{2}\sum_{{v}_{i}, {v}_{j}\in V\left({G}_{1}\right) }\left({{d}_{D}{(v}_{i}, {v}_{j})+d}_{D}{\left({v}_{i}, {v}_{j}\right)}^{2}\right)= \frac{1}{2}\left[505+2017\right]=1261$$
(iii) The domination Harary index of the molecular graph ${G}_{1}$ is obtained by
  $$\begin{aligned}DH\left({G}_{1}\right)&=\frac{1}{2}\sum_{{v}_{i, }{v}_{j} \in V\left({G}_{1}\right)}\frac{1}{{d}_{D}{(v}_{i}, {v}_{j})}\\&=\frac{1}{2}\left[\frac{1}{{d}_{D}({v}_{1},{v}_{2)}}+\frac{1}{{d}_{D}\left({v}_{1},{v}_{3}\right)}+\cdots +\frac{1}{{d}_{D}\left({v}_{1},{v}_{12}\right)}+\frac{1}{{d}_{D}\left({v}_{1},{v}_{13}\right)}+\frac{1}{{d}_{D}\left({v}_{2},{v}_{1}\right)}+\cdots \right. \\ & \quad \left.+\frac{1}{{d}_{D}\left({v}_{2},{v}_{13}\right)}+\cdots +\frac{1}{{d}_{D}({v}_{13} ,{v}_{1)}}+\frac{1}{{d}_{D}\left({v}_{13},{v}_{2}\right)}+\cdots + \frac{1}{{d}_{D}\left({v}_{13}, {v}_{12}\right)}+\frac{1}{{d}_{D}\left({v}_{13}, {v}_{13}\right)}\right]\end{aligned}$$
  $$=\frac{1}{2}\left[\frac{1}{1}+\frac{1}{2}+\cdots +\frac{1}{6}+\frac{1}{5}+\frac{1}{1}+\cdots +\frac{1}{4}+\cdots +\frac{1}{5}+\frac{1}{4}+\cdots +\frac{1}{1}+\frac{1}{1}\right]=36.20238$$
(iv) The domination terminal Weiner index of the molecular graph ${G}_{1}$ is calculated as
  $$DTW\left({G}_{1}\right)=\sum_{{v}_{i, }{v}_{j} \in {V}_{T}\left({G}_{1}\right)}{d}_{D}{(v}_{i}, {v}_{j})$$
  $$=\frac{1}{2}\left[{d}_{D}\left( {v}_{1}, {v}_{3}\right)+{d}_{D}\left({v}_{1}, {v}_{5}\right)+{d}_{D}\left( {v}_{3}, {v}_{5}\right)\right]=\frac{1}{2}\left[2+3+3\right]=8$$
(v) The domination Ashwini index of the molecular graph ${G}_{1}$ is calculated as
  $$DA\left({G}_{1}\right)=\sum_{1\le i<j\le n}{d}_{T}\left( {v}_{i}, {v}_{j}\right)[{\text{deg}}_{T}\left(N\left({v}_{i}\right)\right)+{\text{deg}}_{T}\left(N\left({v}_{j}\right)\right)$$
  $$\begin{aligned} & DA\left( {G_{1} } \right) = d_{T} \left( {v_{1} ,v_{3} } \right)\left[ {\deg _{T} \left( {N\left( {v_{2} } \right)} \right) + \deg _{T} \left( {N\left( {v_{2} } \right)} \right)} \right] \\ &+ d_{T} \left( {v_{1} ,v_{5} } \right)\left[ {\deg _{T} \left( {N\left( {v_{2} } \right)} \right) + \deg _{T} \left( {N\left( {v_{4} } \right)} \right)} \right] + d_{T} \left( {v_{3} ,v_{5} } \right)\deg _{T} \left( {N\left( {v_{2} } \right)} \right) + \deg _{T} \left( {N\left( {v_{4} } \right)} \right) \\ \end{aligned}$$
  $$=2\left(6\right)+3\left(6\right)+3\left(6\right)=48$$
(vi) The domination SM index of the molecular graph ${G}_{1}$ is obtained as
  $$DSM\left({G}_{1}\right)=\sum_{1\le i<j\le n}{d}_{T}\left( {v}_{i}, {v}_{j}\right)\left[{\text{deg}}_{T}\left(N\left({v}_{i}\right)\right)\times {\text{deg}}_{T}\left(N\left({v}_{j}\right)\right)\right]$$
  $$\begin{aligned} DSM\left( {G_{1} } \right) = &\, d_{T} \left( {v_{1} ,v_{3} } \right)\left[ {{\text{deg}}_{T} \left( {N\left( {v_{2} } \right)} \right) \times {\text{deg}}_{T} \left( {N\left( {v_{2} } \right)} \right)} \right] + d_{T} \left( {v_{1} ,v_{5} } \right)\left[ {{\text{deg}}_{T} \left( {N\left( {v_{2} } \right)} \right) \times {\text{deg}}_{T} \left( {N\left( {v_{4} } \right)} \right)} \right] \\ & + d_{T} \left( {v_{3} ,v_{5} } \right)\left[ {{\text{deg}}_{T} \left( {N\left( {v_{2} } \right)} \right) \times {\text{deg}}_{T} \left( {N\left( {v_{4} } \right)} \right)} \right] = 2\left( 9 \right) + 3\left( 9 \right) + 3\left( 9 \right) = 72. \\ \end{aligned}$$

### Theorem 4.2

*Let* $${G}_{2}$$ *be the chemical graph of the drug pyrazinamide. The domination distance based topological indices of the graph*$${G}_{2}$$*are*$$DW\left({G}_{2}\right)=158;$$$$DHW\left({G}_{2}\right)=710;$$$$DH\left({G}_{2}\right)=27.11667;$$$$DSC\left({G}_{2}\right)= 831;$$$$DS{C}^{*}\left({G}_{2}\right)=1053;$$$$DTW\left({G}_{2}\right)=2;$$$$DA\left({G}_{2}\right)=12;$$$$DSM\left({G}_{2}\right)=18$$.

### Proof

**Fig. 3:**
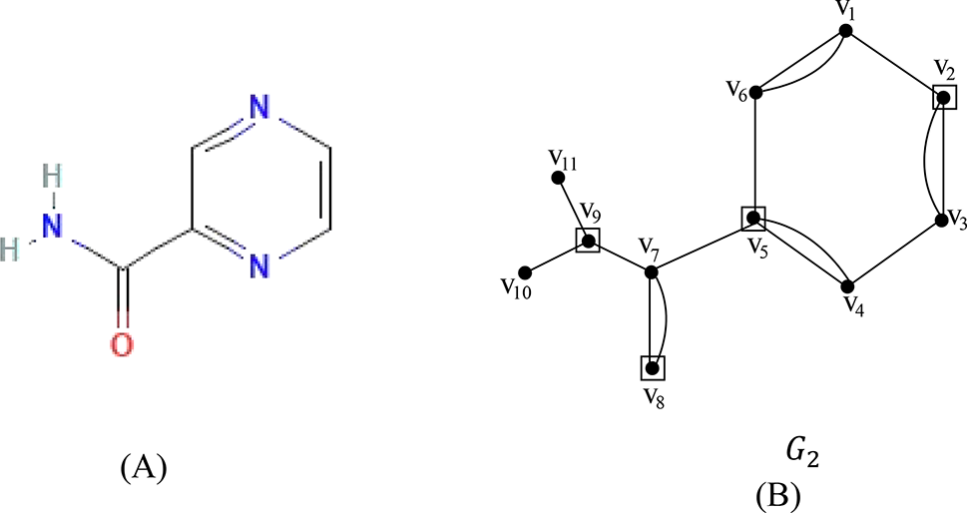
(**A**) Molecular structure of pyrazinamide (**B**) Chemical graph of pyrazinamide.

Let ${G}_{2}$=$\left({V}_{2},{E}_{2}\right)$ be the chemical graph of drug pyrazinamide with 11 atoms (vertices) and 15 bonds (edges) between the atoms. The chemical structure of pyrazinamide and its corresponding chemical graph ${G}_{2}$ are shown in Fig. 3(A) and (B) respectively.

Let ${V}_{2}=\left\{{v}_{1},{v}_{2}, \dots , {v}_{11}\right\}$ be the atom set of ${G}_{2}$. Let the partition of ${V}_{2}$ be ${C}_{1}=\left\{{v}_{1}, {v}_{2}, {v}_{3}, {v}_{4}, {v}_{5},{v}_{6}\right\}$ and ${C}_{2}=\left\{{{v}_{7}, v}_{8}, {v}_{9}, {v}_{10}, {v}_{11}\right\}$ where ${C}_{1}\cup {C}_{2}={V}_{1} \ { {\text{and}}\ C}_{1}\cap {C}_{2}=\varnothing$. The vertex set (atom set) ${V}_{2}$ is partitioned into two subsets ${C}_{1},{C}_{2},$ such that ${C}_{1}$ contains atoms in the benzene ring together with cut edges/bonds and ${C}_{2}$ contains a tree-type structure. In the set ${C}_{1}$ any two non-adjacent atoms in the benzene ring such as $\left\{{v}_{2}, {v}_{5}\right\}$ forms a dominating set and in set ${C}_{2}$ the vertices such as $\left\{{v}_{8},{v}_{9}\right\}$ constitutes the dominating set. The dominating set of ${C}_{1}$ and ${C}_{2}$ are added to get the dominating set of the graph ${G}_{2}$. The dominating set of the graph ${G}_{2}$ is $\left\{{v}_{2},{v}_{5}, {v}_{8}, {v}_{9}\right\}$ and $\gamma \left({G}_{2}\right)= 4$.

The minimum dominating distance matrix of the molecular graph ${G}_{2}$ is obtained using the dominating set of ${G}_{2}$.The minimum dominating distance matrix of ${G}_{2}$ is $D{d}_{ij}\left({G}_{2}\right)$ and is given by

$$D{d}_{ij}\left({G}_{2}\right)=\left(\begin{array}{cccccccccccc}V& {v}_{1}& {v}_{2}& {v}_{3}& {v}_{4}& {v}_{5}& {v}_{6}& {v}_{7}& {v}_{8}& {v}_{9}& {v}_{10}& {v}_{11}\\ {v}_{1}& 0& 1& 2& 3& 2& 1& 3& 4& 4& 5& 5\\ {v}_{2}& 1& 0& 1& 2& 3& 2& 4& 5& 5& 6& 6\\ {v}_{3}& 2& 1& 0& 1& 2& 3& 3& 4& 4& 5& 5\\ {v}_{4}& 3& 2& 1& 0& 1& 2& 2& 3& 3& 4& 4\\ {v}_{5}& 2& 3& 2& 1& 0& 1& 1& 2& 2& 3& 3\\ {v}_{6}& 1& 2& 3& 2& 1& 0& 2& 3& 3& 4& 4\\ {v}_{7}& 3& 4& 3& 2& 1& 2& 0& 1& 1& 2& 2\\ {v}_{8}& 4& 5& 4& 3& 2& 3& 1& 0& 2& 3& 3\\ {v}_{9}& 4& 5& 4& 3& 2& 3& 1& 2& 0& 1& 1\\ {v}_{10}& 5& 6& 5& 4& 3& 4& 2& 3& 1& 0& 2\\ {v}_{11}& 5& 6& 5& 4& 3& 4& 2& 3& 1& 2& 0\end{array}\right)$$

The degree of each vertex of the molecular graph ${G}_{2}$ is calculated and are tabulated in Table 2.

**Table 2:** The degree of the vertices of the molecular graph ${G}_{2}$.

Vertex	${v}_{1}$	${v}_{2}$	${v}_{3}$	${v}_{4}$	${v}_{5}$	${v}_{6}$	${v}_{7}$	${v}_{8}$	${v}_{9}$	${v}_{10}$	${v}_{11}$
Degree	3	3	3	3	4	3	4	2	3	1	1

For every pair of vertices ${v}_{i}, {v}_{j}$ of the vertex set ${V}_{2}$, the summation values of $\left(d{v}_{i}+d{v}_{j}\right)$ and ($d{v}_{i}\times d{v}_{j})$ of pyrazinamide are computed from the minimum dominating distance matrix and degree of vertices and are summarized in Table 3.

**Table 3:** The values $\sum_{{v}_{i}, {v}_{j} \in {G}_{2}}\left(d{v}_{i}+d{v}_{j}\right)$ and $\sum_{{v}_{i}, {v}_{j} \in { G}_{2}}\left(d{v}_{i}\times d{v}_{j}\right)$ with different distances of the molecular graph ${G}_{2}$ of pyrazinamide.

The distance $d\left({v}_{i}, {v}_{j}\right)$	1	2	3	4	5	6
$\sum_{{v}_{i}, {v}_{j} \in {G}_{2}}\left(d{v}_{i}+d{v}_{j}\right)$	182	164	142	90	54	16
$\sum_{{v}_{i}, {v}_{j} \in {G}_{2}}\left(d{v}_{i}\times d{v}_{j}\right)$	280	238	192	108	54	12

Substituting the values of Tables 2 and 3 in Eqs. (1) and (2), the domination Schultz polynomial and domination modified Schultz polynomial of the drug pyrazinamide are given by

5 $$DSC\left({G}_{2}, x\right)=91x+{82x}^{2}+71{x}^{3}+45{x}^{4}+27{x}^{5}+8{x}^{6}$$

6 $$DS{C}^{*}\left({G}_{2},x\right)=140x+119{x}^{2}+96 {x}^{3}+54 {x}^{4}+27 {x}^{5}+6 {x}^{6}$$

The domination Schultz index and domination modified Schultz index are obtained from Eqs. (5) and (6) respectively.

The domination Schultz index $$\left(DSC\right)$$ = $${\left.\frac{\partial \left(DSC\left({G}_{2}, x\right)\right)}{\partial x}\right|}_{x=1}$$ and therefore $$\frac{\partial \left(DSC\left({G}_{2}, x\right)\right)}{\partial x}=$$$$91+164x+213{x}^{2}+180{x}^{3}+135{x}^{4}+48{x}^{5}$$ and hence $$\left[{\left.\frac{\partial \left(DSC\left({G}_{2}, x\right)\right)}{\partial x}\right|}_{x=1}\right]$$ = 831.

The domination modified Schultz index $$\left(DS{C}^{*}\right)$$ = $${\left.\frac{\partial \left(DS{C}^{*}\left({G}_{2}, x\right)\right)}{\partial x}\right|}_{x=1}$$ and therefore $$\frac{\partial \left(DS{C}^{*}\left({G}_{2}, x\right)\right)}{\partial x}=$$$$140+238 x+288 {x}^{2}+216 {x}^{3}+135 {x}^{4}+36 {x}^{5}$$ and hence $$\left[{\left.\frac{\partial \left(DS{C}^{*}\left({G}_{2}, x\right)\right)}{\partial x}\right|}_{x=1}\right]$$ = 1053.

The domination distance based topological indices are computed using minimum dominating distance matrix $D{d}_{ij}\left({G}_{2}\right)$ for the drug pyrazinamide and are as follows:

(i) The domination Weiner index of the molecular graph ${G}_{2}$ is computed as
  $$DW\left({G}_{2}\right)=\frac{1}{2}\sum_{{v}_{i, }{v}_{j} \in V\left({G}_{2}\right)}{d}_{D}{(v}_{i}, {v}_{j})=158$$
(ii) The domination hyper Weiner index of the molecular graph ${G}_{2}$ is calculated as
  $$DHW\left({G}_{2}\right)=\frac{1}{2}\sum_{{v}_{i}, {v}_{j}\in V\left({G}_{2}\right) }\left({{d}_{D}{(v}_{i}, {v}_{j})+d}_{D}{\left({v}_{i}, {v}_{j}\right)}^{2}\right)=710$$
(iii) The domination Harary index of the molecular graph ${G}_{2}$ is obtained by
  $$DH\left({G}_{2}\right)=\frac{1}{2}\sum_{{v}_{i, }{v}_{j} \in V\left({G}_{2}\right)}\frac{1}{{d}_{D}{(v}_{i}, {v}_{j})} =27.1161$$
(iv) The domination terminal Weiner index of the molecular graph ${G}_{2}$ is calculated as
  $$DTW\left({G}_{2}\right)=\sum_{{v}_{i, }{v}_{j} \in {V}_{T}\left({G}_{2}\right)}{d}_{D}{(v}_{i}, {v}_{j})=2$$
(v) The domination Ashwini index of the molecular graph ${G}_{2}$ is computed as
  $$DA\left({G}_{2}\right)=\sum_{1\le i<j\le n}{d}_{T}\left( {v}_{i}, {v}_{j}\right)\left[{\text{deg}}_{T}\left(N\left({v}_{i}\right)\right)+{\text{deg}}_{T}\left(N\left({v}_{j}\right)\right) \right]=12$$
(vi) The domination SM index of the molecular graph ${G}_{2}$ is calculated as
  $$DSM\left({G}_{2}\right)=\sum_{1\le i<j\le n}{d}_{T}\left( {v}_{i}, {v}_{j}\right)\left[{\text{deg}}_{T}\left(N\left({v}_{i}\right)\right)\times {\text{deg}}_{T}\left(N\left({v}_{j}\right)\right)\right] =18.$$

### Theorem 4.3

*Let* $${G}_{3}$$ *be the chemical graph of the drug ethambutol. The domination distance based topological indices of the graph*$${G}_{3}$$*are*$$DW\left({G}_{3}\right)=708;$$$$DHW\left({G}_{3}\right)=4944;$$$$DH\left({G}_{3}\right)=53.9798;$$$$DSC\left({G}_{3}\right)= 2546;$$$$DS{C}^{*}\left({G}_{3}\right)=1537;$$$$DTW\left({G}_{3}\right)=206;$$$$DA\left({G}_{3}\right)=470;$$$$DSM\left({G}_{3}\right)=537$$.

### Proof

**Fig. 4:**
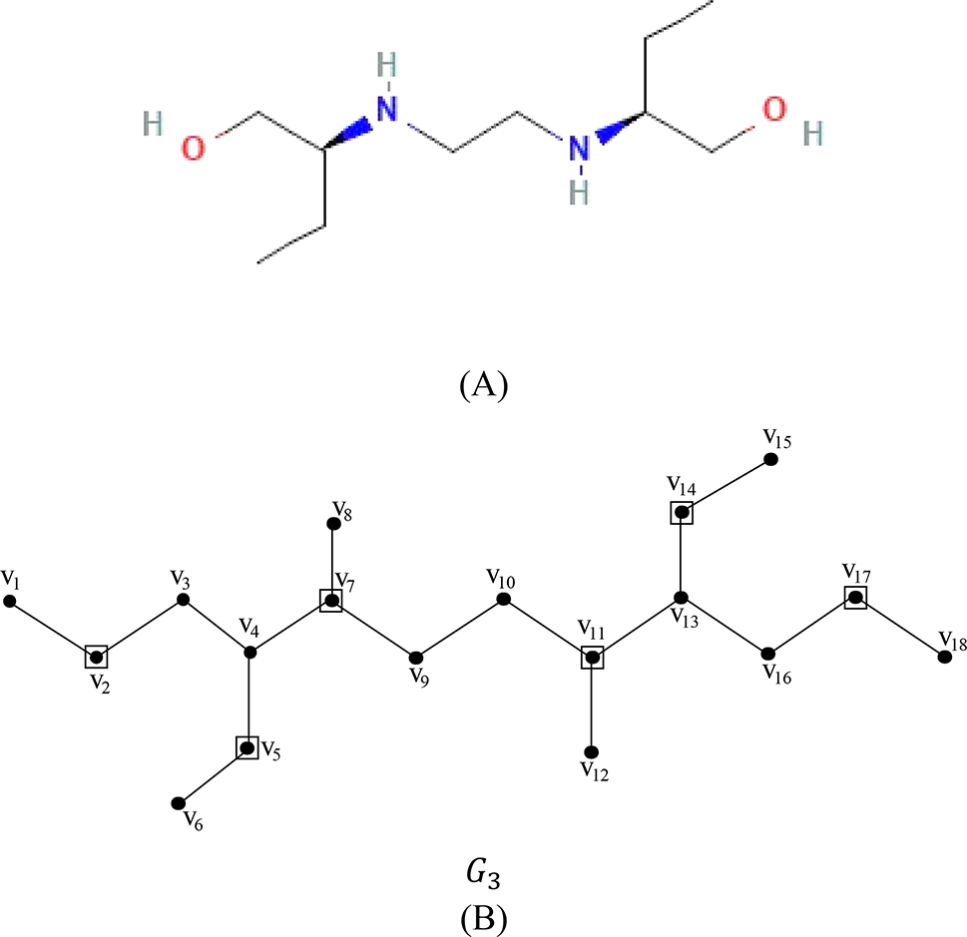
(**A**) Chemical structure of ethambutol (**B**) Chemical graph of ethambutol.

Let $${G}_{3}$$$$=\left({V}_{3},{E}_{3}\right)$$ be the chemical graph of drug ethambutol with 18 atoms (vertices) and 17 bonds (edges) between the atoms. The chemical structure of ethambutol and its corresponding chemical graph $${G}_{3}$$ are shown in Fig. 4(A) and (B) respectively.

Let ${V}_{3}=\left\{{v}_{1},{v}_{2}, \dots , {v}_{18}\right\}$ be the atom set of ${G}_{3}$. The vertex ${v}_{2}$ dominates the vertices ${v}_{1}$ and ${v}_{3}$ since ${v}_{2}$ is adjacent to ${v}_{1}$ and ${v}_{3}$. The vertex ${v}_{5}$ dominates the vertices ${v}_{4}$ and ${v}_{6}$ since ${v}_{5}$ is adjacent to ${v}_{4}$ and ${v}_{6}$. The vertex ${v}_{7}$ dominates the vertices ${v}_{8}$ and ${v}_{9}$ since ${v}_{7}$ is adjacent to ${v}_{8}$ and ${v}_{9}$. The vertex ${v}_{11}$ dominates the vertices ${v}_{10}$ and ${v}_{12}$ since ${v}_{11}$ is adjacent to ${v}_{10}$ and ${v}_{12}$. The vertex ${v}_{14}$ dominates the vertices ${v}_{13}$ and ${v}_{15}$ since ${v}_{14}$ is adjacent to ${v}_{13}$ and ${v}_{15}$. The vertex ${v}_{17}$ dominates the vertices ${v}_{16}$ and ${v}_{18}$ since ${v}_{17}$ is adjacent to ${v}_{16}$ and ${v}_{18}$. Hence the vertices $\left\{{v}_{2},{v}_{5}, {v}_{7}, {v}_{11}, {v}_{14}, {v}_{17}\right\}$ forms a dominating set. The domination number of the graph ${G}_{3}$ is $\gamma \left({G}_{3}\right)$ = 6.

The minimum dominating distance matrix of the molecular graph ${G}_{3}$ is calculated using the dominating set of ${G}_{3}$. The minimum dominating distance matrix $D{d}_{ij}\left({G}_{3}\right)$ is as follows.
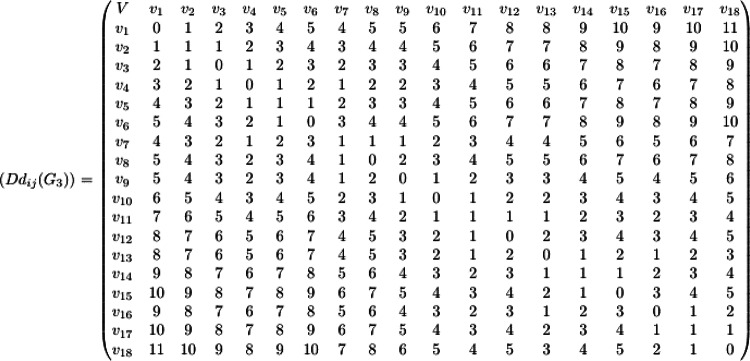


The degree of each vertex of the molecular graph ${G}_{3}$ is calculated and are tabulated in Table 4.

**Table 4:** The degree of each vertex of the molecular graph ${G}_{3}$.

Vertex	${v}_{1}$	${v}_{2}$	${v}_{3}$	${v}_{4}$	${v}_{5}$	${v}_{6}$	${v}_{7}$	${v}_{8}$	${v}_{9}$	${v}_{10}$	${v}_{11}$	${v}_{12}$	${v}_{13}$	${v}_{14}$	${v}_{15}$	${v}_{16}$	${v}_{17}$	${v}_{18}$
Degree	1	2	2	3	2	1	3	1	2	2	3	1	3	2	1	2	2	1

For every pair of vertices ${v}_{i}, {v}_{j}$ of the vertex set ${V}_{3}$, the summation values of $\left(d{v}_{i}+d{v}_{j}\right)$ and ($d{v}_{i}\times d{v}_{j})$ of ethambutol are computed from the minimum dominating distance matrix and degree of vertices and are summarized in Table 5.

**Table 5:** The values $\sum_{{v}_{i}, {v}_{j} \in {G}_{3}}\left(d{v}_{i}+d{v}_{j}\right)$ and $\sum_{{v}_{i}, {v}_{j} \in {G}_{3}}\left(d{v}_{i}\times d{v}_{j}\right)$ of the molecular graph ${G}_{3}$ of ethambutol (Distances 1,2,…,11).

Distance $d\left({v}_{i}, {v}_{j}\right)$	1	2	3	4	5	6	7	8	9	10	11
$\frac{1}{2}\sum_{{v}_{i}, {v}_{j} \in {G}_{3}}\left(d{v}_{i}+d{v}_{j}\right)$	102	86	90	80	64	56	52	40	24	10	4
$\frac{1}{2}\sum_{{v}_{i}, {v}_{j} \in {G}_{3}}\left(d{v}_{i}\times d{v}_{j}\right)$	114	88	85	72	60	54	46	32	17	6	1

Using the values of Tables 4 and 5 and substituting the values in Eqs. 1 and 2, the domination Schultz polynomial and domination modified Schultz polynomial of the drug ethambutol are given by,

7 $$DSC\left({G}_{3}, x\right)=102x+{86x}^{2}+90{x}^{3}+80{x}^{4}+64{x}^{5}+56{x}^{6}+ 52{x}^{7}+{40x}^{8}+{24x}^{9}+{10x}^{10}+{4 x}^{11}$$

8 $$DS{C}^{*}\left({G}_{3}, x\right)=114x+{88x}^{2}+85{x}^{3}+72{x}^{4}+60{x}^{5}+54{x}^{6}+ 46{x}^{7}+{32x}^{8}+{17x}^{9}+{6x}^{10}+{x}^{11}$$

The domination Schultz index and domination modified Schultz index are obtained from Eqs. (7) and (8) respectively and are given as $\left[{\left.\frac{\partial \left(DSC\left({G}_{3}, x\right)\right)}{\partial x}\right|}_{x=1}\right] =2546$ and $\left[{\left.\frac{\partial \left(DS{C}^{*}\left({G}_{3}, x\right)\right)}{\partial x}\right|}_{x=1}\right] = 1537$.

The domination distance based topological indices are computed using minimum dominating distance matrix $D{d}_{ij}\left({G}_{3}\right)$ for the drug ethambutol and are given as follows:

(i) The domination Weiner index of the molecular graph ${G}_{3}$ is computed as
  $$DW\left({G}_{3}\right)=\frac{1}{2}\sum_{{v}_{i, }{v}_{j} \in V\left({G}_{3}\right)}{d}_{D}{(v}_{i}, {v}_{j})=708$$
(ii) The domination hyper Weiner index of the molecular graph ${G}_{3}$ is calculated as
  $$DHW\left({G}_{3}\right)=\frac{1}{2}\sum_{{v}_{i}, {v}_{j}\in V\left({G}_{3}\right) }\left({{d}_{D}{(v}_{i}, {v}_{j})+d}_{D}{\left({v}_{i}, {v}_{j}\right)}^{2}\right)=4944$$
(iii) The domination Harary index of the molecular graph ${G}_{3}$ is obtained as
  $$DH\left({G}_{3}\right)=\frac{1}{2}\sum_{{v}_{i, }{v}_{j} \in V\left({G}_{3}\right)}\frac{1}{{d}_{D}{(v}_{i}, {v}_{j})}=53.9798$$
(iv) The domination terminal Weiner index of the molecular graph ${G}_{3}$ is computed as
  $$DTW\left({G}_{3}\right)=\sum_{{v}_{i, }{v}_{j} \in {V}_{T}\left({G}_{3}\right)}{d}_{D}{(v}_{i}, {v}_{j})=206$$
(v) The domination Ashwini index of the molecular graph ${G}_{3}$ is calculated as
  $$DA\left({G}_{3}\right)=\sum_{1\le i<j\le n}{d}_{T}\left( {v}_{i}, {v}_{j}\right)\left[{\text{deg}}_{T}\left(N\left({v}_{i}\right)\right)+{\text{deg}}_{T}\left(N\left({v}_{j}\right)\right)\right] =470$$
(vi) The domination SM index of the molecular graph ${G}_{3}$ is obtained as
  $$DSM\left({G}_{3}\right)=\sum_{1\le i<j\le n}{d}_{T}\left( {v}_{i}, {v}_{j}\right)\left[{\text{deg}}_{T}\left(N\left({v}_{i}\right)\right)\times {\text{deg}}_{T}\left(N\left({v}_{j}\right)\right)\right] =537.$$

### Theorem 4.4

*Let* $${G}_{4}$$ *be the chemical graph of the drug ethionamide. The domination distance based topological indices of the graph*$${G}_{4}$$*are*$$DW\left({G}_{4}\right)=252.5;$$$$DHW\left({G}_{4}\right)=1261;$$$$DH\left({G}_{4}\right)=36.20238;$$$$DSC\left({G}_{4}\right)= 1235;$$$$DS{C}^{*}\left({G}_{4}\right)=1464;$$$$DTW\left({G}_{4}\right)=8;$$$$DA\left({G}_{4}\right)=48;$$$$DSM\left({G}_{4}\right)=72.$$

### Proof

**Fig. 5:**
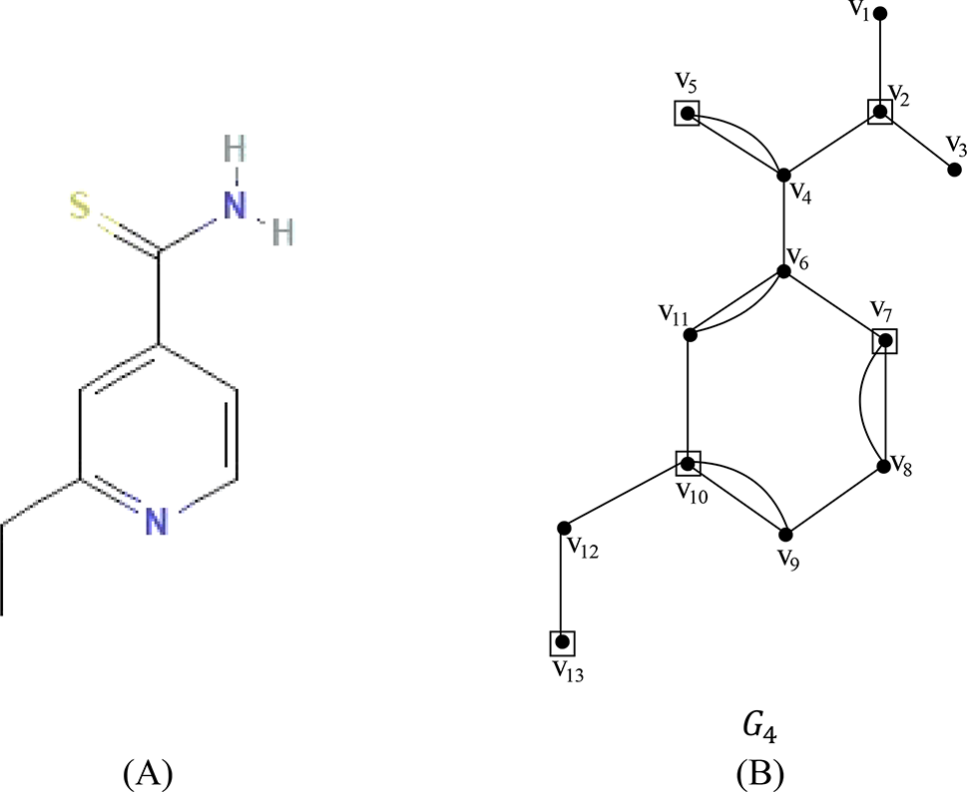
(**A**) Chemical structure of ethionamide (**B**) Chemical graph of ethionamide.

Let ${G}_{4}=\left({V}_{4},{E}_{4}\right)$ be the chemical graph of the drug ethionamide with 13 atoms(vertices) and 17 bonds (edges) between the atoms. The chemical structure of ethionamide and its corresponding chemical graph ${G}_{4}$ are shown in Fig. 5(A) and (B) respectively.

Let ${V}_{4}=\left\{{v}_{1}, {v}_{2}, {v}_{3},\dots , {v}_{12},{v}_{13}\right\}$ be the atom set of ${G}_{4}$. Let the partition of ${V}_{4}$ be ${C}_{1}=\left\{{v}_{1}, {v}_{2}, {v}_{3}, {v}_{4} , {v}_{5},{v}_{12}, {v}_{13}\right\}$ and ${C}_{2}= \left\{{{v}_{6}, {v}_{7}, v}_{8}, {v}_{9}, {v}_{10}, {v}_{11}\right\},$ where ${C}_{1}\cup {C}_{2}={V}_{1}{, C}_{1}\cap {C}_{2}=\varnothing$.

The vertex set (atom set) ${V}_{4}$ is partitioned into two subsets ${C}_{1},{C}_{2},$ such that ${C}_{1}$ contains a set of pendant vertices and adjacent atoms linked with the pendant vertices and ${C}_{2}$ contains atoms in the benzene ring together with cut edges/bonds. In the set ${C}_{1}$ the vertices $\left\{{v}_{2}, {v}_{5}, {v}_{13}\right\}$ constitutes a dominating set and in set ${C}_{2}$ any two non-adjacent atoms in the benzene ring $\left\{{v}_{7}, {v}_{10}\right\}$ forms the dominating set. The dominating set of ${C}_{1}$ and ${C}_{2}$ are added to get the dominating set of the graph ${G}_{4}$. The dominating set of the graph ${G}_{4}$ is $\left\{{v}_{2},{v}_{5}, {v}_{7}, {v}_{10}, {v}_{13}\right\}$ and therefore the domination number of ${G}_{4}$ is $\gamma \left({G}_{4}\right)= 5$.

The minimum dominating distance matrix of the molecular graph ${G}_{4}$ is obtained using the dominating set of ${G}_{4}$. The minimum dominating distance matrix of ${G}_{4}$ is $D{d}_{ij}\left({G}_{4}\right)$ and is given by

$$D{d}_{ij}\left({G}_{4}\right)=\left(\begin{array}{cccccccccccccc}V& {v}_{1}& {v}_{2}& {v}_{3}& {v}_{4}& {v}_{5}& {v}_{6}& {v}_{7}& {v}_{8}& {v}_{9}& {v}_{10}& {v}_{11}& {v}_{12}& {v}_{13}\\ {v}_{1}& 0& 1& 2& 2& 3& 3& 4& 5& 6& 5& 4& 6& 7\\ {v}_{2}& 1& 1& 1& 1& 2& 2& 3& 4& 5& 4& 3& 5& 6\\ {v}_{3}& 2& 1& 0& 2& 3& 3& 4& 5& 6& 5& 4& 6& 7\\ {v}_{4}& 2& 1& 2& 0& 1& 1& 2& 3& 4& 3& 2& 4& 5\\ {v}_{5}& 3& 2& 3& 1& 1& 2& 3& 4& 5& 4& 3& 5& 6\\ {v}_{6}& 3& 2& 3& 1& 2& 0& 1& 2& 3& 2& 1& 3& 4\\ {v}_{7}& 4& 3& 4& 2& 3& 1& 1& 1& 2& 3& 2& 4& 5\\ {v}_{8}& 5& 4& 5& 3& 4& 2& 1& 0& 1& 2& 3& 3& 4\\ {v}_{9}& 6& 5& 6& 4& 4& 3& 2& 1& 0& 1& 2& 2& 3\\ {v}_{10}& 5& 4& 5& 3& 5& 2& 3& 2& 1& 1& 1& 1& 2\\ {v}_{11}& 4& 3& 4& 2& 6& 1& 2& 3& 2& 1& 0& 2& 3\\ {v}_{12}& 6& 5& 6& 4& 5& 3& 4& 3& 2& 1& 2& 0& 1\\ {v}_{13}& 7& 6& 7& 5& 4& 4& 5& 4& 3& 2& 3& 1& 1\end{array}\right)$$

For every pair of vertices ${v}_{i}, {v}_{j}$ of the vertex set ${V}_{4}$, the summation values of $(d{v}_{i}+d{v}_{j})$ and ($d{v}_{i}\times d{v}_{j})$ of ethionamide are computed from the minimum dominating distance matrix and degree of vertices and are summarized in Table 7.

Substituting the values of Tables 6 and 7 in Eqs. (1) and (2), the domination Schultz polynomial and domination modified Schultz polynomial of the drug ethionamide are given by

**Table 6:** The degree of the vertices of the molecular graph ${G}_{4}$.

Vertex	${v}_{1}$	${v}_{2}$	${v}_{3}$	${v}_{4}$	${v}_{5}$	${v}_{6}$	${v}_{7}$	${v}_{8}$	${v}_{9}$	${v}_{10}$	${v}_{11}$	${v}_{12}$	${v}_{13}$
Degree	1	3	1	4	2	4	3	3	3	4	3	2	1

**Table 7:** The values $\sum_{{v}_{i}, {v}_{j} \in {G}_{4}}(d{v}_{i}+d{v}_{j})$ and $\sum_{{v}_{i}, {v}_{j} \in {G}_{4}}(d{v}_{i}\times d{v}_{j})$ with different distances of the chemical graph ${G}_{4}$ of ethionamide.

The distance $d({v}_{i}, {v}_{j})$	1	2	3	4	5	6	7
$\frac{1}{2}\sum_{{v}_{i}, {v}_{j} \in {G}_{4}}(d{v}_{i}+d{v}_{j})$	104	99	92	67	47	21	4
$\frac{1}{2}\sum_{{v}_{i}, {v}_{j} \in {G}_{4}}(d{v}_{i}\times d{v}_{j})$	157	142	123	80	46	15	2

9 $$DSC({G}_{4}, x)=104x+99{x}^{2}+92{x}^{3}+67{x}^{4}+47{x}^{5}+21{x}^{6}+4{x}^{7}$$

10 $$DS{C}^{*}\left({G}_{4},x\right)=157x+142{x}^{2}+123 {x}^{3}+80 {x}^{4}+46 {x}^{5}+15 {x}^{6}+2{x}^{7}$$

The domination Schultz index and domination modified Schultz index are obtained from Eqs. (9) and (10) respectively and are given as $\left[{\left.\frac{\partial \left(DSC\left({G}_{4}, x\right)\right)}{\partial x}\right|}_{x=1}\right] =1235$ and $\left[{\left.\frac{\partial \left(DS{C}^{*}\left({G}_{4}, x\right)\right)}{\partial x}\right|}_{x=1}\right] = 1464$.

The domination distance based topological indices calculated using the dominating distance matrix of ethionamide are as follows:

(i) The domination Weiner index of the molecular graph ${G}_{4}$ is computed as
  $$DW\left({G}_{4}\right)=\frac{1}{2}\sum_{{v}_{i, }{v}_{j} \in V\left({G}_{3}\right)}{d}_{D}{(v}_{i}, {v}_{j})=252.5$$
(ii) The domination hyper Weiner index of the molecular graph ${G}_{4}$ is calculated as
  $$DHW\left({G}_{4}\right)=\frac{1}{2}\sum_{{v}_{i}, {v}_{j}\in V\left({G}_{4}\right) }\left({{d}_{D}{(v}_{i}, {v}_{j})+d}_{D}{\left({v}_{i}, {v}_{j}\right)}^{2}\right)=1261$$
(iii) The domination Harary index of the molecular graph ${G}_{4}$ is obtained as
  $$DH\left({G}_{4}\right)=\frac{1}{2}\sum_{{v}_{i, }{v}_{j} \in V\left({G}_{4}\right)}\frac{1}{{d}_{D}{(v}_{i}, {v}_{j})}=30.20238$$
(iv) The domination terminal Weiner index of the molecular graph ${G}_{4}$ is computed as
  $$DTW\left({G}_{4}\right)=\sum_{{v}_{i, }{v}_{j} \in {V}_{T}\left({G}_{4}\right)}{d}_{D}{(v}_{i}, {v}_{j})=16$$
(v) The domination Ashwini index of the molecular graph ${G}_{4}$ is calculated as
  $$DA\left({G}_{4}\right)=\sum_{1\le i<j\le n}{d}_{T}\left( {v}_{i}, {v}_{j}\right)\left[{\text{deg}}_{T}\left(N\left({v}_{i}\right)\right)+{\text{deg}}_{T}\left(N\left({v}_{j}\right)\right)\right] =82$$
(vi The domination SM index of the molecular graph ${G}_{4}$ is obtained as
  $$DSM\left({G}_{4}\right)=\sum_{1\le i<j\le n}{d}_{T}\left( {v}_{i}, {v}_{j}\right)\left[{\text{deg}}_{T}\left(N\left({v}_{i}\right)\right)\times {\text{deg}}_{T}\left(N\left({v}_{j}\right)\right)\right] =102.$$

### Theorem 4.5

*Let* $${G}_{5}$$ *be the chemical graph of the drug linezolid. The domination distance based topological indices of the graph*$${G}_{5}$$*are*$$DW\left({G}_{5}\right)=1632;$$$$DHW\left({G}_{5}\right)=13630 ;$$$$DSC\left({G}_{5}\right)=7993;$$$$DS{C}^{*}\left({G}_{5}\right)=9717;$$$$DTW\left({G}_{5}\right)=22;$$$$DA\left({G}_{5}\right)=164;$$$$DSM\left({G}_{5}\right)=304;$$$$DH\left({G}_{5}\right)=187.9864$$

### Proof

**Fig. 6:**
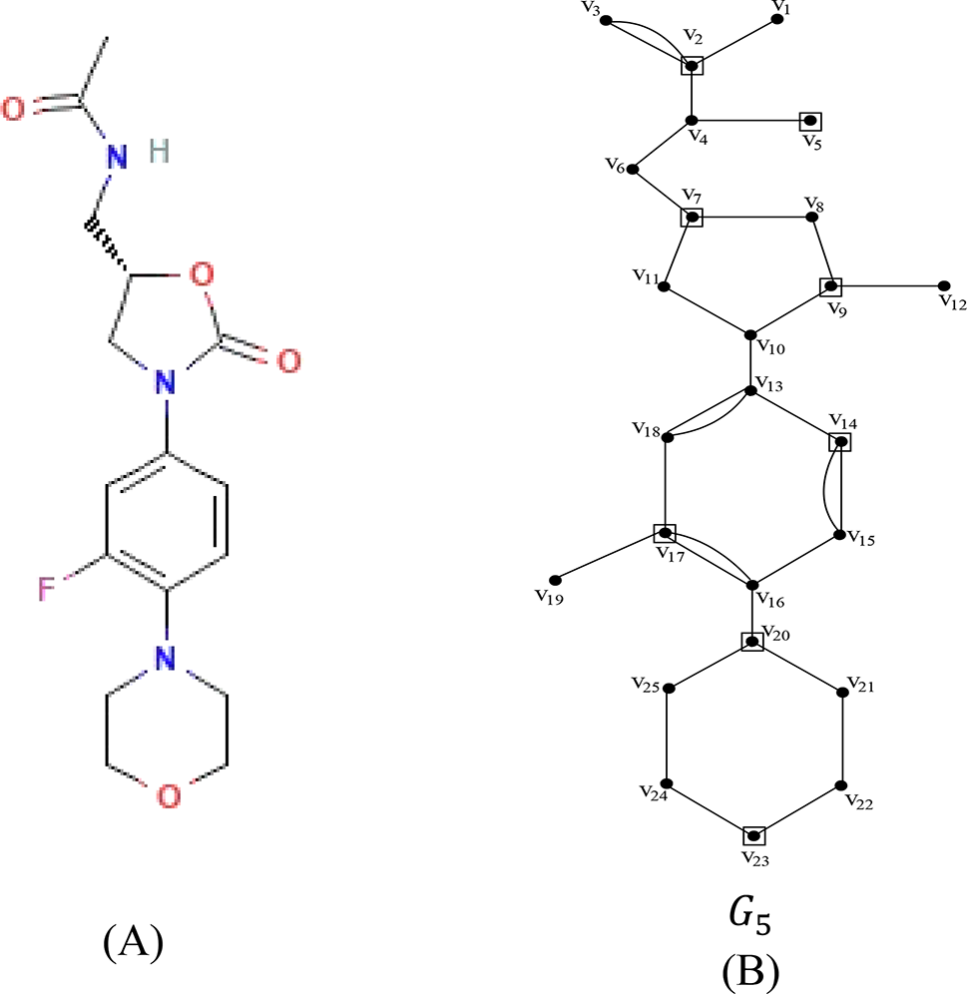
(**A**) Chemical structure of linezolid (**B**) Chemical graph of linezolid.

Let ${G}_{5}=\left({V}_{5},{E}_{5}\right)$ be the chemical graph of the drug linezolid with 25 atoms(vertices) and 32 bonds (edges) between the atoms. The chemical structure of linezolid and its corresponding chemical graph ${G}_{5}$ are shown in Fig. 6(A) and (B) respectively.

Let ${V}_{5}=\left\{{v}_{1},{v}_{2}, \dots , {v}_{25}\right\}$ be the atom set of ${G}_{5}$. Let the partition ${V}_{5}$ of ${G}_{5}$ be ${C}_{1}= \left\{{v}_{1}, {v}_{2}, {v}_{3}, {v}_{4} , {v}_{5}\right\}$; ${C}_{2}= \left\{{{v}_{6}, v}_{7}, {v}_{8}, {v}_{9}, {v}_{10}, {v}_{11}, {v}_{12}\right\}$;${ C}_{3}= \left\{{v}_{13}, {v}_{14}, {v}_{15}, {v}_{16}, {v}_{17}, {v}_{18}, {v}_{19}\right\}$ and ${C}_{4}= \left\{{v}_{20}, {v}_{21}, {v}_{22}, {v}_{23}, {v}_{24}, {v}_{25}\right\}$ where ${C}_{1}\cup {C}_{2 }\cup {C}_{3}\cup {C}_{4}={V}_{5} \ { {\text{and}} \ C}_{1}\cap {C}_{2}\cap {C}_{3}\cap {C}_{4}=\varnothing$. The vertex set (atom set) ${V}_{5}$ is partitioned into four subsets ${C}_{1},{C}_{2}, {C}_{3}, {C}_{4}$ such that ${C}_{1}$ contains a set of pendant atoms and adjacent atoms linked with the pendant atoms and ${C}_{2}, {C}_{3}, {C}_{4}$ contains atoms in the benzene ring together with cut edges/bonds. In the set ${C}_{1}$ the atom with maximum degree and the pendant atom not linked with the atom that contains maximum degree such as $\left\{{v}_{2},{v}_{5}\right\}$ constitutes the dominating set and in the set ${C}_{2 ,}{C}_{3}$ and ${C}_{4}$ any two non-adjacent atoms in each benzene ring such as $\left\{{v}_{7}, {v}_{9}, {v}_{14}, {v}_{17}, {v}_{20},{v}_{23}\right\}$ forms the dominating set. The dominating set of each partition is calculated and added to get the dominating set of the graph ${G}_{5}$. Hence, the dominating set of the graph ${G}_{5}$ is $\left\{{{v}_{2}, {v}_{5}, v}_{7}, {v}_{9}, {v}_{14}, {v}_{17}, {v}_{20},{v}_{23}\right\}$ and therefore the domination number of the graph is $\gamma \left({G}_{5}\right)= 8$.

The domination distance based topological indices are computed using minimum dominating distance matrix $D{d}_{ij}\left({G}_{5}\right)$ for the drug linezolid and are given as follows:
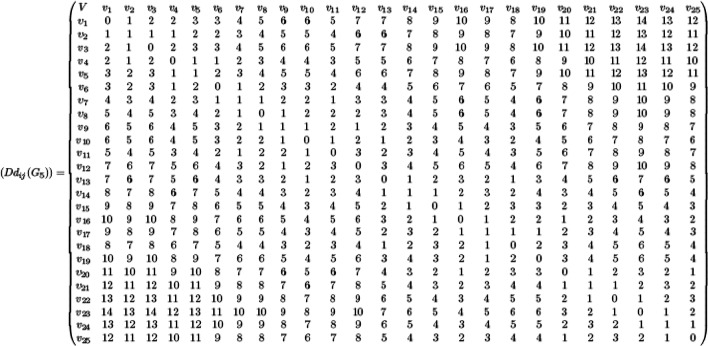


For every pair of vertices ${v}_{i}, {v}_{j}$ of the vertex set ${V}_{5}$, the summation values of $(d{v}_{i}+d{v}_{j})$ and ($d{v}_{i}\times d{v}_{j})$ of linezolid are computed from the minimum dominating distance matrix and degree of vertices and are summarized in Tables 9 and 10.

Substituting the values of Tables 8, 9 and 10 in Eqs. (1) and (2), the domination Schultz polynomial and domination modified Schultz polynomial of the drug linezolid are given by

**Table 8:** The degree of the vertices of the molecular graph ${G}_{5}$.

Vertex	${v}_{1}$	${v}_{2}$	${v}_{3}$	${v}_{4}$	${v}_{5}$	${v}_{6}$	${v}_{7}$	${v}_{8}$	${v}_{9}$	${v}_{10}$	${v}_{11}$	${v}_{12}$	${v}_{13}$	${v}_{14}$
Degree	1	4	3	3	1	2	3	2	4	3	2	2	4	3
Vertex	${v}_{15}$	${v}_{16}$	${v}_{17}$	${v}_{18}$	${v}_{19}$	${v}_{20}$	${v}_{21}$	${v}_{22}$	${v}_{23}$	${v}_{24}$	${v}_{25}$			
Degree	3	4	4	3	1	3	2	2	2	2	2

**Table 9:** The values $\sum_{{v}_{i}, {v}_{j} \in {G}_{5}}(d{v}_{i}+d{v}_{j})$ and $\sum_{{v}_{i}, {v}_{j} \in {G}_{5}}(d{v}_{i}\times d{v}_{j})$ of the molecular graph ${G}_{5}$ of linezolid (Distances 1, 2, 3, …, 7).

Distance $d({v}_{i}, {v}_{j})$	1	2	3	4	5	6	7
$\frac{1}{2}\sum_{{v}_{i}, {v}_{j} \in {G}_{5}}(d{v}_{i}+d{v}_{j})$	199	205	202	184	172	141	114
$\frac{1}{2}\sum_{{v}_{i}, {v}_{j} \in {G}_{5}}(d{v}_{i}\times d{v}_{j})$	289	278	268	240	223	178	144

**Table 10:** The values $\sum_{{v}_{i}, {v}_{j} \in {G}_{5}}(d{v}_{i}+d{v}_{j})$ and $\sum_{{v}_{i}, {v}_{j} \in {G}_{5}}(d{v}_{i}\times d{v}_{j})$ of the molecular graph ${G}_{5}$ of linezolid (Distances 8, 9, 10, …,14).

Distance $d({v}_{i}, {v}_{j})$	8	9	10	11	12	13	14
$\frac{1}{2}\sum_{{v}_{i}, {v}_{j} \in {G}_{5}}(d{v}_{i}+d{v}_{j})$	109	90	58	41	37	23	7
$\frac{1}{2}\sum_{{v}_{i}, {v}_{j} \in {G}_{5}}(d{v}_{i}\times d{v}_{j})$	132	100	64	45	38	22	6

11 $$DSC\left({G}_{5}, x\right)=199x+{205x}^{2}+202{x}^{3}+184{x}^{4}+172{x}^{5}+141{x}^{6}+ 114{x}^{7}+{109x}^{8}+{90x}^{9}+{58x}^{10}+{4 1x}^{11}+37{x}^{12}+23{x}^{13}+7{x}^{14}$$

12 $$DS{C}^{*}\left({G}_{5}, x\right)=289x+{278x}^{2}+268{x}^{3}+240{x}^{4}+223{x}^{5}+178{x}^{6}+ 144{x}^{7}+{132x}^{8}+{100x}^{9}+{64x}^{10}+{4 5x}^{11}+38{x}^{12}+22{x}^{13}+6{x}^{14}$$

The domination Schultz index and domination modified Schultz index are obtained from Eqs. (11) and (12) respectively and are given as $\left[{\left.\frac{\partial \left(DSC\left({G}_{5}, x\right)\right)}{\partial x}\right|}_{x=1}\right] =7993$ and $\left[{\left.\frac{\partial \left(DS{C}^{*}\left({G}_{5}, x\right)\right)}{\partial x}\right|}_{x=1}\right] = 9717$.

The domination distance based topological indices are computed using minimum dominating distance matrix $D{d}_{ij}\left({G}_{5}\right)$ for the drug linezolid and are given as follows

(i) The domination Weiner index of the molecular graph ${G}_{5}$ is computed as
  $$DW\left({G}_{5}\right)=\frac{1}{2}\sum_{{v}_{i, }{v}_{j} \in V\left({G}_{5}\right)}{d}_{D}{(v}_{i}, {v}_{j})=1632$$
(ii) The domination hyper Weiner index of the molecular graph ${G}_{5}$ is computed as
  $$DHW\left({G}_{5}\right)=\frac{1}{2}\sum_{{v}_{i}, {v}_{j}\in V\left({G}_{5}\right) }\left({{d}_{D}{(v}_{i}, {v}_{j})+d}_{D}{\left({v}_{i}, {v}_{j}\right)}^{2}\right)=13630$$
(iii) The domination Harary index of the molecular graph ${G}_{5}$ is obtained as
  $$DH\left({G}_{5}\right)=\frac{1}{2}\sum_{{v}_{i, }{v}_{j} \in V\left({G}_{5}\right)}\frac{1}{{d}_{D}{(v}_{i}, {v}_{j})}=93.99321$$
(iv) The domination terminal Weiner index of the molecular graph ${G}_{5}$ is computed as
  $$DTW\left({G}_{5}\right)=\sum_{{v}_{i, }{v}_{j} \in {V}_{T}\left({G}_{5}\right)}{d}_{D}{(v}_{i}, {v}_{j})=22$$
(v) The domination Ashwini index of the molecular graph ${G}_{5}$ is computed as
  $$DA\left({G}_{5}\right)=\sum_{1\le i<j\le n}{d}_{T}\left( {v}_{i}, {v}_{j}\right)\left[{\text{deg}}_{T}\left(N\left({v}_{i}\right)\right)+{\text{deg}}_{T}\left(N\left({v}_{j}\right)\right)\right]=164$$
(vi) The domination SM index of the molecular graph ${G}_{5}$ is computed as.
  $$DSM\left({G}_{5}\right)=\sum_{1\le i<j\le n}{d}_{T}\left( {v}_{i}, {v}_{j}\right)\left[{\text{deg}}_{T}\left(N\left({v}_{i}\right)\right)\times {\text{deg}}_{T}\left(N\left({v}_{j}\right)\right)\right]=304.$$

### Theorem 4.6

*Let* $${G}_{6}$$ *be the chemical graph of the drug levofloxacin. The domination distance based topological indices of the graph*$${G}_{6}$$*are*$$DW\left({G}_{6}\right)=1666.5; DHW\left({G}_{6}\right)=11849;$$$$DSC\left({G}_{6}\right)= 8386;$$$$DS{C}^{*}\left({G}_{6}\right)=10381;$$$$DTW\left({G}_{6}\right)=52;$$$$DA\left({G}_{6}\right)=278;$$$$DSM\left({G}_{6}\right)=442;$$$$DH\left({G}_{6}\right)=114.163$$

### Proof

**Fig. 7:**
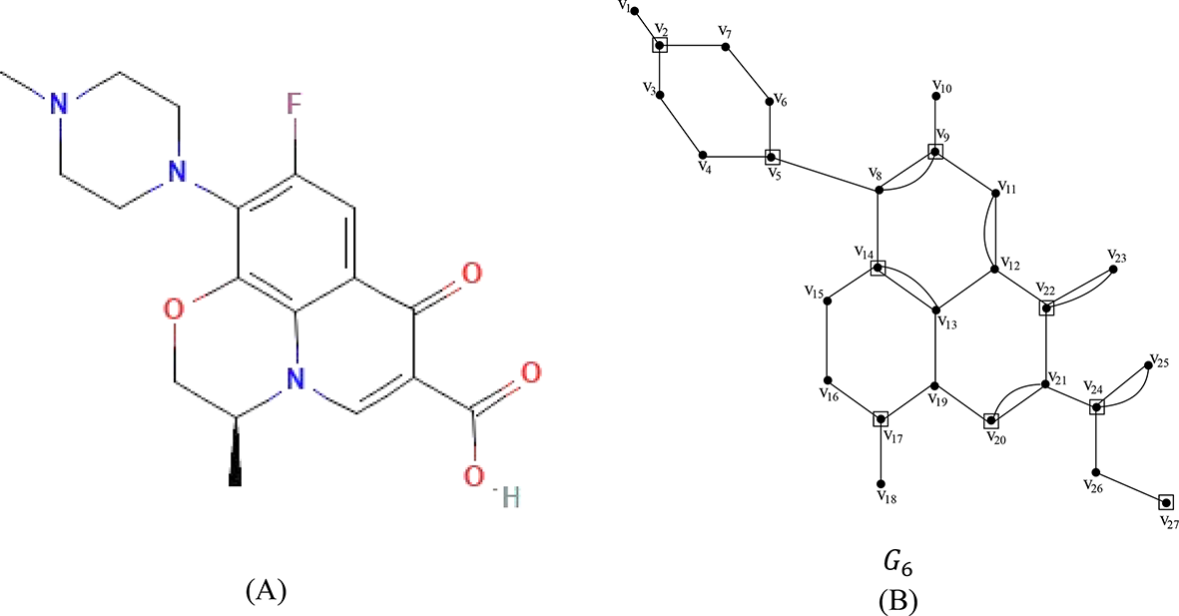
(**A**) Chemical structure of levofloxacin (**B**) Chemical graph of levofloxacin.

Let ${G}_{6}=\left({V}_{6},{E}_{6}\right)$ be the chemical graph of the drug levofloxacin with 27 atoms(vertices) and 36 bonds (edges) between the atoms. The chemical structure of levofloxacin and its corresponding chemical graph are shown in Fig. 7(A) and (B) respectively.

Let ${V}_{6}=\left\{{v}_{1},{v}_{2}, \dots , {v}_{27}\right\}$ be the atom set of ${G}_{6}$. Let the partition of ${V}_{6}$ be ${C}_{1}= \left\{{v}_{1}, {v}_{2}, {v}_{3}, {v}_{4} , {v}_{5},{v}_{6}\right\}$ and ${C}_{2}= \left\{{v}_{8}, {v}_{9}, {v}_{10}, {v}_{11}, {v}_{12}, {{v}_{13}, v}_{14}, {v}_{15}, {v}_{16}, {v}_{17}, {v}_{18}, {v}_{19}, {v}_{20}, {v}_{21}, {v}_{22}, {v}_{23}\right\}$ and ${C}_{3}= \left\{ {v}_{24}, {v}_{25}, {v}_{26}, {v}_{27}\right\},$ where ${C}_{1}\cup {C}_{2 }\cup {C}_{3}={V}_{6} \ { \text{and } \  C}_{1}\cap {C}_{2}\cap {C}_{3}=\varnothing$. The set ${C}_{1}$ contains atoms in the benzene ring together with cut edges/bonds, ${C}_{2}$ contains atoms with adjacent benzene rings linked together and ${C}_{3}$ contains remaining set of atoms of ${G}_{6}$. In the set ${C}_{1}$ any two non-adjacent atoms in the benzene ring, such as $\left\{{v}_{2}, {v}_{5}\right\}$ constitutes a dominating set and in set ${C}_{2}$ any two non-adjacent atoms in each benzene ring such as $\left\{{v}_{9}, {v}_{14},{v}_{17}, {v}_{20}, {v}_{22}\right\}$ constitutes the dominating set and in set ${C}_{3}$ the vertex ${v}_{24}$ dominates the vertices ${v}_{25}$ and ${v}_{26}$ and the vertex ${v}_{27}$ dominates itself. The dominating set of ${C}_{3}$ is $\left\{{v}_{24},{v}_{27}\right\}$. The dominating set of each set is added and hence the dominating set of the graph ${G}_{6}$ is $\left\{{{v}_{2}, {v}_{5},v}_{9}, {v}_{14},{v}_{17}, {v}_{20}, {v}_{22},{v}_{24}, {v}_{27}\right\}.$ The domination number of the graph ${G}_{6}$ is 9 and hence $\gamma \left({G}_{6}\right)= 9$.

The minimum dominating distance matrix of the molecular graph ${G}_{6}$ is obtained using the dominating set of ${G}_{6}$ .The minimum dominating distance matrix of ${G}_{6}$ is $D{d}_{ij}\left({G}_{6}\right)$ and is given by.
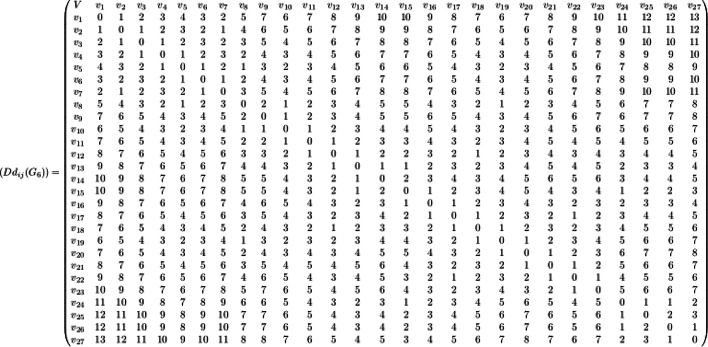


For every pair of vertices ${v}_{i}, {v}_{j}$ of the vertex set ${V}_{6}$, the summation values of $(d{v}_{i}+d{v}_{j})$ and ($d{v}_{i}\times d{v}_{j})$ of ${G}_{6}$ are computed from the minimum dominating distance matrix and degree of vertices and are summarized in Table 11.

**Table 11:** The degree of each vertex of the molecular graph ${G}_{6}$.

$\text{Vertex}$	${v}_{1}$	${v}_{2}$	${v}_{3}$	${v}_{4}$	${v}_{5}$	${v}_{6}$	${v}_{7}$	${v}_{8}$	${v}_{9}$	${v}_{10}$	${v}_{11}$	${v}_{12}$	${v}_{13}$	${v}_{14}$
$\text{Degree}$	1	3	2	2	3	2	2	4	4	1	3	4	4	4
$\text{Vertex}$	${v}_{15}$	${v}_{16}$	${v}_{17}$	${v}_{18}$	${v}_{19}$	${v}_{20}$	${v}_{21}$	${v}_{22}$	${v}_{23}$	${v}_{24}$	${v}_{25}$	${v}_{26}$	${v}_{27}$	
$\text{Degree}$	2	2	3	1	3	3	4	4	2	4	2	2	1	

Substituting the values of Tables 11, 12 and 13 in Eqs. (1) and (2), the domination Schultz polynomial and domination modified Schultz polynomial of the drug levofloxacin are given by

**Table 12:** The values $\sum_{{v}_{i}, {v}_{j} \in {G}_{6}}(d{v}_{i}+d{v}_{j})$ and $\sum_{{v}_{i}, {v}_{j} \in {G}_{6}}(d{v}_{i}\times d{v}_{j})$ of the molecular graph ${G}_{6}$ of levofloxacin(Distances 1,2,3, …,7).

Distance $d({v}_{i}, {v}_{j})$	1	2	3	4	5	6	7
$\frac{1}{2}\sum_{{v}_{i}, {v}_{j} \in {G}_{6}}(d{v}_{i}+d{v}_{j})$	230	269	310	293	258	200	140
$\frac{1}{2}\sum_{{v}_{i}, {v}_{j} \in {G}_{6}}(d{v}_{i}\times d{v}_{j})$	357	405	447	399	329	233	157

**Table 13:** The values $\sum_{{v}_{i}, {v}_{j} \in {G}_{6}}(d{v}_{i}+d{v}_{j})$ and $\sum_{{v}_{i}, {v}_{j} \in {G}_{6}}(d{v}_{i}\times d{v}_{j})$ of the molecular graph ${G}_{6}$ of levofloxacin (Distances 8, 9,…,14).

Distance $d({v}_{i}, {v}_{j})$	8	9	10	11	12	13
$\frac{1}{2}\sum_{{v}_{i}, {v}_{j} \in {G}_{6}}(d{v}_{i}+d{v}_{j})$	89	63	39	21	10	2
$\frac{1}{2}\sum_{{v}_{i}, {v}_{j} \in {G}_{6}}(d{v}_{i}\times d{v}_{j})$	102	68	39	20	7	1

13 $$DSC\left({G}_{6}, x\right)=230x+{269x}^{2}+310{x}^{3}+293{x}^{4}+258{x}^{5}+200{x}^{6}+ 140{x}^{7}+{89x}^{8}+{63x}^{9}+{39x}^{10}+{2 1x}^{11}+10{x}^{12}+2{x}^{13}$$

14 $$DS{C}^{*}\left({G}_{6}, x\right)=357x+{405x}^{2}+447{x}^{3}+399{x}^{4}+329{x}^{5}+233{x}^{6}+ 157{x}^{7}+{102x}^{8}+{68x}^{9}+{39x}^{10}+{20x}^{11}+7{x}^{12}+{x}^{13}$$

The domination Schultz index and modified domination Schultz index are obtained from Eqs. (13) and (14) respectively and are given as $\left[{\left.\frac{\partial \left(DSC\left({G}_{6}, x\right)\right)}{\partial x}\right|}_{x=1}\right] =10381$ and $\left[{\left.\frac{\partial \left(DS{C}^{*}\left({G}_{6}, x\right)\right)}{\partial x}\right|}_{x=1}\right] = 8386$

The domination distance based topological indices are computed using minimum dominating distance matrix $D{d}_{ij}\left({G}_{6}\right)$ for the drug levofloxacin and are given as follows

(i) The domination Weiner index of the molecular graph ${G}_{6}$ is computed as
  $$DW\left({G}_{6}\right)=\frac{1}{2}\sum_{{v}_{i, }{v}_{j} \in V\left({G}_{6}\right)}{d}_{D}{(v}_{i}, {v}_{j})=1666.5$$
(ii) The domination hyper Weiner index of the molecular graph ${G}_{6}$ is computed as
  $$DHW\left({G}_{6}\right)=\frac{1}{2}\sum_{{v}_{i}, {v}_{j}\in V\left({G}_{6}\right) }\left({{d}_{D}{(v}_{i}, {v}_{j})+d}_{D}{\left({v}_{i}, {v}_{j}\right)}^{2}\right)=11849$$
(iii) The domination Harary index of the molecular graph ${G}_{6}$ is obtained as
  $$DH\left({G}_{6}\right)=\frac{1}{2}\sum_{{v}_{i, }{v}_{j} \in V\left({G}_{6}\right)}\frac{1}{{d}_{D}{(v}_{i}, {v}_{j})}=114.163$$
(iv) The domination terminal Weiner index of the molecular graph ${G}_{6}$ is computed as
  $$DTW\left({G}_{6}\right)=\sum_{{v}_{i, }{v}_{j} \in {V}_{T}\left({G}_{6}\right)}{d}_{D}{(v}_{i}, {v}_{j})=52$$
(v) The domination Ashwini index of the molecular graph ${G}_{6}$ is computed as
  $$DA\left({G}_{6}\right)=\sum_{1\le i<j\le n}{d}_{T}\left( {v}_{i}, {v}_{j}\right)\left[{\text{deg}}_{T}\left(N\left({v}_{i}\right)\right)+{\text{deg}}_{T}\left(N\left({v}_{j}\right)\right)\right]=278$$
(vi) The domination SM index of the molecular graph ${G}_{6}$ is computed as
  $$DSM\left({G}_{6}\right)=\sum_{1\le i<j\le n}{d}_{T}\left( {v}_{i}, {v}_{j}\right)\left[{\text{deg}}_{T}\left(N\left({v}_{i}\right)\right)\times {\text{deg}}_{T}\left(N\left({v}_{j}\right)\right)\right]=442.$$

The computed domination distance-based topological indices for the molecular graph of isoniazid, pyrazinamide, ethambutol, ethionamide, linezolid and levofloxacin are tabulated in Table 14.

**Table 14:** Computed domination distance-based topological indices for tuberculosis treatment drugs.

Drugs	DW(G)	DHW(G)	DTW(G)	DH(G)	DSC(G)	DSC*(G)	DA(G)	DSM(G)
Isoniazid	252.5	1261	8	36.2023	1271	1537	48	72
Pyrazinamide	158	710	2	27.1167	831	1053	12	18
Ethambutol	708	4944	206	53.9798	2546	1537	470	537
Ethionamide	252.5	1261	16	35.2024	1235	1464	82	102
Linezolid	1632	13,630	22	93.9932	7993	9717	164	304
Levofloxacin	1666.5	11,849	52	114.163	8386	10,381	278	442

DW- Domination Weiner index; DHW- Domination Hyper Weiner index; DTW- Domination Terminal Weiner index; DH- Domination Harary index; DSC- Domination Schultz index; DSC*- Domination modified Schultz index; DA- Domination Ashwini index; DSM- Domination SM index.

## QSPR analysis for physicochemical properties of tuberculosis treatment drugs

### Methodology

The QSPR analysis is carried out for the drugs isoniazid, pyrazinamide, ethambutol, ethionamide, linezolid and levofloxacin using eight domination distance - based topological indices. The quadratic regression^36^ is employed to explore the physicochemical properties of the drugs used to treat tuberculosis. The quadratic regression equation

$$Z=A+B\left(X\right)+C{\left(X\right)}^{2}$$

is considered for analyzing the physicochemical properties of the drugs. Here Z is the dependent variable representing the physicochemical properties of the drug, $A$ is a constant, $B$ and $C$ are the regression coefficients and $X$ represent independent variables which are different domination distance-based indices obtained through minimum dominating distance matrix. The regression analysis is performed using SPSS software (https://www.ibm.com/spss). The analysis is carried over, ten physicochemical properties viz., Boiling point (BP), Melting point (MP), Flash point (FP), Molar refraction (MR), Enthalpy of vaporization (EV), Polarizability (P), Molar volume (MV), log P (LOGP), Molar weight (MW) and Polar surface area (SA). The physicochemical properties of the tuberculosis treating drugs considered for QSPR analysis are taken from the database www.ChemSpider.com and are summarized in Table 15.

**Table 15:** Physicochemical properties of tuberculosis treatment drugs.

Drugs	Properties									
	BP	MP	FP	MR	EV	P	MV	MW	LOGP	SA
Isoniazid	251.97	172	251	36.9	–	14.6	110.2	137.142	− 0.3149	58.037
Pyrazinamide	173.3	190	119.1	31.9	54.1	12.6	87.7	123.115	− 0.4245	51.737
Ethambutol	345.3	89	113.7	58.6	68.3	23.2	207	204.314	− 0.2926	86.712
Ethionamide	247.9	− 163	103.7	49	46.5	19.4	142	166.249	1.2782	71.445
Linezolid	585.5	177	307.9	83	87.5	32.9	259	337.351	1.1236	138.854
Levofloxacin	571.5	224	299.4	91.1	90.1	36.1	244	361.373	1.544	148.732

Boiling Point-BP, Melting Point -MP, Flash Point-FP, Molar Refraction-MR, Enthalpy of Vaporization-EV, Polarizability- P, Molar Volume-MV, Molar Weight- MW, Log P -LOGP, Polar Surface Area-SA.

### Results and discussion

The quadratic regression analysis is carried out to examine the relationship between the physicochemical properties (dependent variables) and the domination distance-based topological indices (independent variables). The results of this analysis are represented by the square of the correlation coefficient ${R}^{2}$, and are presented in Table 16. The highly correlated indices with the physicochemical properties of the drugs in terms of ${R}^{2}$ are highlighted in bold letters in the Table 16.

**Table 16:** The ${R}^{2}$ values between computed topological indices and physicochemical properties of the tuberculosis treatment drugs.

Indices	Properties									
	BP	MP	FP	MR	EV	P	MV	MW	LOGP	SA
$DW$	0.987	0.929	0.690	0.955	**0.958**	0.955	0.971	0.985	0.514	0.981
$DHW$	0.983	0.471	0.663	0.941	0.956	0.941	0.956	0.962	0.432	0.960
$DTW$	0.694	0.872	0.499	0.830	0.572	0.830	0.704	0.783	0.756	0.789
$DH$	0.986	0.823	0.598	**0.969**	0.933	**0.969**	0.971	0.984	0.500	**0.983**
$DSC$	**0.994**	**0.976**	0.683	0.955	0.945	0.955	**0.976**	**0.987**	0.527	**0.983**
$DS{C}^{*}$	0.954	0.589	**0.723**	0.875	0.849	0.875	0.786	0.943	0.529	0.934
$DA$	0.890	0.685	0.598	0.958	0.695	0.958	0.915	0.914	**0.830**	0.923
$DSM$	0.909	0.347	0.561	0.913	0.714	0.913	0.976	0.868	0.671	0.878

The quadratic regression analysis is the best suited for predicting physicochemical properties, based on its ability to generate maximum R-squared ${(R}^{2})$ values, along with p-values indicating significance level is below 0.05 when compared to other regression models.

The quadratic equations having highest correlation coefficient ${R}^{2}$ and the statistical parameter such as minimum standard error (SE), maximum (R) value, maximum F-value, and p-value less than 0.05 are summarized in Table 17.

**Table 17:** The statistical parameters of highly correlated indices with physicochemical properties of the tuberculosis treatment drugs.

Quadratic Equation	R	F	SE	p
$BP = 96.27+0.12\left(DSC\right)-7.61E-6{\left(DSC\right)}^{2}$	0.997	255.871	17.345	0.000
$MP=-2.72E2-0.1\left(DSC\right)+1.12E-5{(DSC)}^{2}$	0.988	61.163	8.921	0.004
$MR=1.38+1.3\left(DH\right)-4.57E-3{(DH)}^{2}$	0.985	47.492	5.466	0.005
$EV=43.88+0.04\left(DW\right)-5.15E-6{(DW)}^{2}$	0.979	22.763	5.647	0.042
$P=0.48+0.5\left(DH\right)-1.82E-3{(DH)}^{2}$	0.985	47.508	2.169	0.005
$MV=16.95+0.1\left(DSC\right)-8.2E-6{(DSC)}^{2}$	0.988	61.399	14.307	0.004
$SA=37.32+0.02\left(DSC\right)-1.1E-6{(DSC)}^{2}$	0.991	85.654	7.039	0.002
$MW=88.88+0.05\left(DSC\right)-2.29E-6{(DSC)}^{2}$	0.993	110.896	15.374	0.002
$MV=53.2+1.1\left(DSM\right)-1.51E-3{(DSM)}^{2}$	0.988	61.061	14.346	0.004
$SA=4.03+1.87\left(DH\right)-51.5E-3{(DH)}^{2}$	0.992	87.266	6.975	0.002

**Fig. 8:**
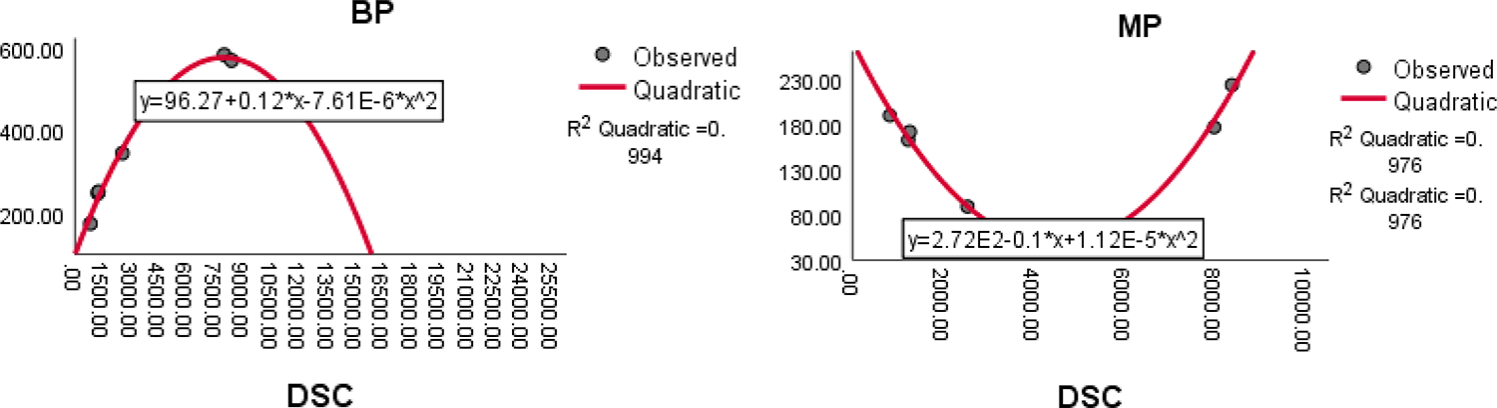
The quadratic regression curve of domination Schultz index $(DSC)$ against Boiling point $(BP)$ and melting point $(MP)$.

**Fig. 9:**
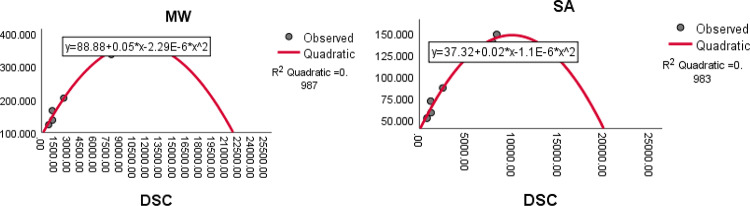
The quadratic regression curve of domination Schultz index $(DSC)$ against Molecular weight *(MW) *and Surface area *(SA)*.

**Fig. 10:**
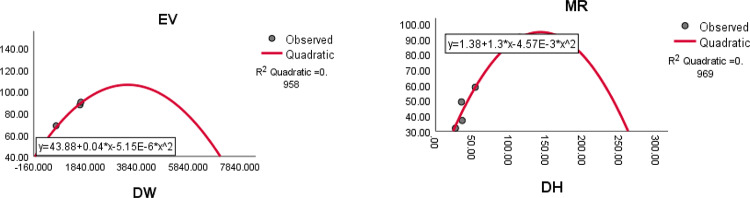
The quadratic regression curve of domination Weiner index $(DW)$ and domination Harary index $(DH)$ with Enthalpy of vaporization *(EV)* and Molar Refraction *(MR)*.

**Fig. 11:**
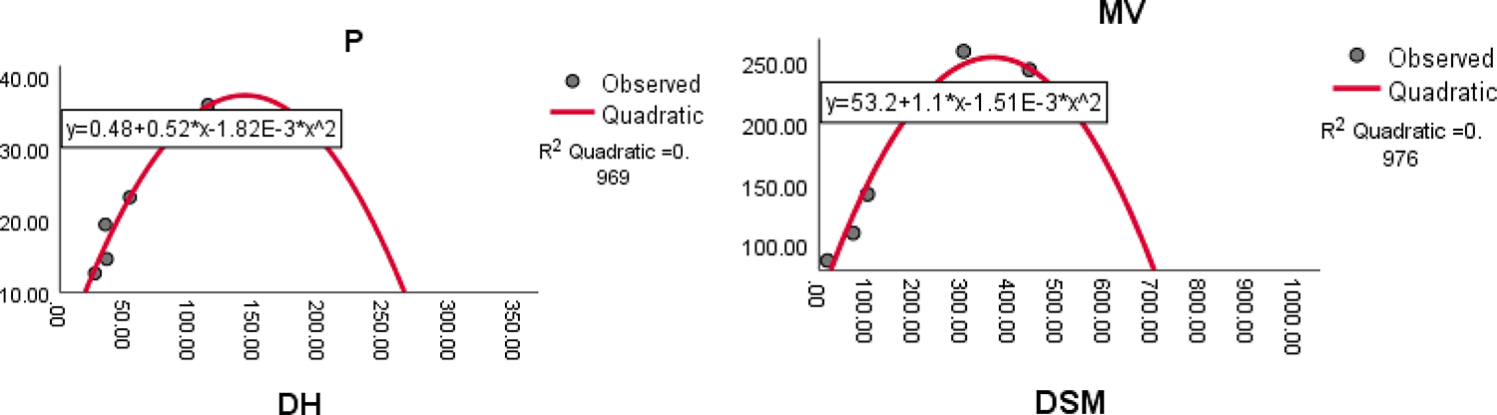
The quadratic regression curve of domination Harary index $(DH)$ and domination SM index $(DSM)$ against polarization $(P)$ and molar volume *(MV)*.

The quadratic regression curve plots for the most reliable domination index having highest ${R}^{2}$ with $p<0.05$ and the physicochemical properties of antituberculosis drugs are shown in Figs. 8, 9, 10 and 11.

Based on the statistical parameters of highly correlated indices with the properties and their regression plots, the following results are obtained.

(i) The domination Schultz index (DSC) is most suitable for predicting the boiling point (BP), melting point (MP), molar volume (MV), molar weight (MW) and polar surface area (PSA).
(ii) The domination Harary index (DH) is most suitable for predicting the molar refraction (R), polarizability (P) and polar surface area (PSA)
(iii) The domination Weiner index (DW) is most suitable for predicting the enthalpy of vaporisation (EV).
(iv) The domination modified Schultz index (DSC*) is most suitable for predicting the flash point (FP).
(v) The domination SM index (DSM) most suitable for predicting the molar volume. (MV).

## QSPR analysis for ADMET properties of tuberculosis treatment drugs

### Methodology

The quadratic regression equation provided in Sect. "Methodology" is considered in the QSPR analysis to explore the ADMET properties of the tuberculosis treatment drugs. The analysis is carried over, ten ADMET properties viz., Water Solubility (WS), Intestinal absorption (IA), Fraction Unbound (FU), BBB Permeability (BBB), CNS Permeability (CNS), Total Clearance (TC), Maximum Tolerated Dose (MTD), Oral Rat Acute Toxicity (AT), Oral Rat Chronic Toxicity (CT), and T. pyriformis (TPY). The regression analysis is performed using the SPSS software tool (https://www.ibm.com/spss). The ADMET properties of the drugs are obtained from web tool pkCSM and are summarized in Table 18.

**Table 18:** ADMET properties of tuberculosis treatment drugs.

Drugs	Properties									
	WS	IA	FU	BBB	$\text{CNS}$	$\text{TC}$	$\text{AT}$	MTD	CT	TPY
Isoniazid	− 1.6	92.601	0.728	0.002	− 3.351	0.722	2.304	1.166	1.395	− 0.314
Pyrazinamide	− 0.615	92.813	0.773	− 0.013	− 2.972	0.666	2.047	1.354	2.714	− 0.482
Ethambutol	− 0.967	66.168	0.851	− 0.21	− 3.555	1.234	1.895	0.987	2.637	− 0.362
Ethionamide	− 1.958	99.428	0.582	− 0.265	− 2.837	0.035	2.498	0.902	1.258	0.084
Linezolid	− 3.097	90.415	0.297	− 0.755	− 3.08	0.231	2.863	0.046		0.593
Levofloxacin	− 3.179	97.397	0.577	− 0.792	− 3.054	0.414	2.59	0.965	1.791	0.285

Water Solubility-WS, Intestinal absorption-IA, Fraction Unbound-FU, BBB Permeability- BBB, CNS Permeability -CNS, Total Clearance -TC, Maximum Tolerated Dose -MTD, Oral Rat Acute Toxicity-AT, Oral Rat Chronic Toxicity -CT, T. pyriformis- TPY.

### Results and discussion

The results of the analysis, represented by the square of the correlation co-efficient ${R}^{2}$, are presented in Table 19. The highly correlated indices with the ADMET properties of the drugs in terms of ${R}^{2}$ are highlighted in bold letters in the Table 19. The quadratic equations with the highest correlation coefficient ${R}^{2}$ and the statistical parameter such as minimal standard error (SE), maximum (R) value, maximum F-value, and p significance value less than 0.05 are tabulated in the Table 20.

**Table 19:** The ${R}^{2}$ values between computed topological indices and ADMET properties of the tuberculosis treatment drugs.

Indices	Properties									
	$\text{WS}$	IA	FU	BBB	$\text{CNS}$	$\text{TC}$	$\text{AT}$	MTD	CT	TPY
$DW$	0.789	0.837	0.606	0.932	**0.618**	0.511	0.658	0.486	0.178	0.680
$DHW$	0.744	0.549	0.845	0.905	0.471	0.538	0.763	0.651	0.303	0.767
$DTW$	0.813	**0.934**	0.461	0.775	0.595	0.692	0.679	0.132	0.406	0.592
$DH$	0.739	0.535	0.376	0.910	0.375	0.166	0.404	0.635	0.075	0.585
$DSC$	0.796	0.814	0.552	**0.935**	0.607	0.442	0.609	0.528	0.136	0.672
$DS{C}^{*}$	0.858	0.179	0.711	0.925	0.173	0.165	0.674	**0.880**	0.407	0.851
$DA$	**0.990**	0.879	0.761	0.927	0.579	**0.799**	0.931	0.435	0.619	0.899
$DSM$	0.911	0.675	**0.930**	0.862	0.460	0.771	**0.980**	0.763	**0.805**	**0.996**

**Table 20:** The statistical parameters of highly correlated indices with ADMET properties of the tuberculosis treatment drugs.

Quadratic Equation	R	F	SE	p
$WS = -0.44-0.22\left(DA\right)+4.75E-5{\left(DA\right)}^{2}$	0.995	148.808	0.138	0.001
$IA= 92.43+0.15\left(DTW\right)-1.36E-3{\left(DTW\right)}^{2}$	0.967	21.330	3.988	0.017
$FU= 0.91-4.09E-3\left(DSM\right)+7.43E-6{\left(DSM\right)}^{2}$	0.964	19.951	0.067	0.018
$BBB= 0.02-9.62E-5\left(DSC\right)-7.52E-11{\left(DSC\right)}^{2}$	0.967	21.496	0.116	0.017
$AT= 1.87+7.57E-3\left(DSM\right)-1.39E-5{\left(DSM\right)}^{2}$	0.990	73.522	0.065	0.003
$MTD=2.93-1.49E-3 \left(DS{C}^{*}\right)+1.24E-7{\left(DS{C}^{*}\right)}^{2}$	0.938	10.987	0.202	0.042
$TPY=-0.66+8.82E-3 \left(DSM\right)-1.53E-5{\left(DSM\right)}^{2}$	0.998	339.435	0.035	0.000

**Fig. 12:**
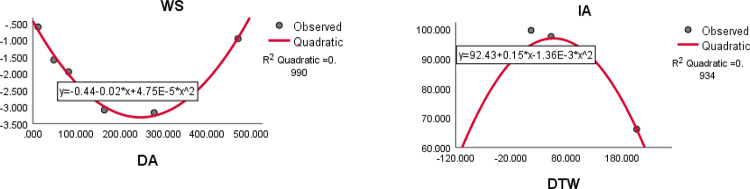
The quadratic regression curve of domination Ashwini index and domination terminal Weiner index $(DA)$ against water solubility *(WS)* and intestinal absorption $(IA)$.

**Fig. 13:**
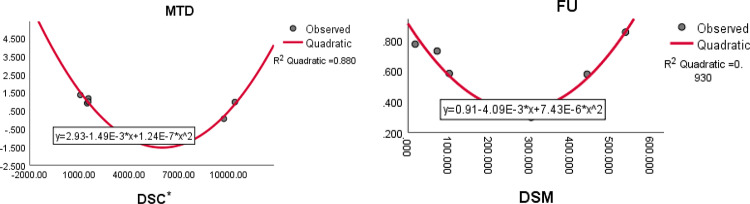
The quadratic regression curve of domination modified Schultz index $(DS{C}^{*})$ and domination SM index $(DSM)$ against maximum tolerated dose *(MTD)* and fraction unbound *(FU)*.

**Fig. 14:**
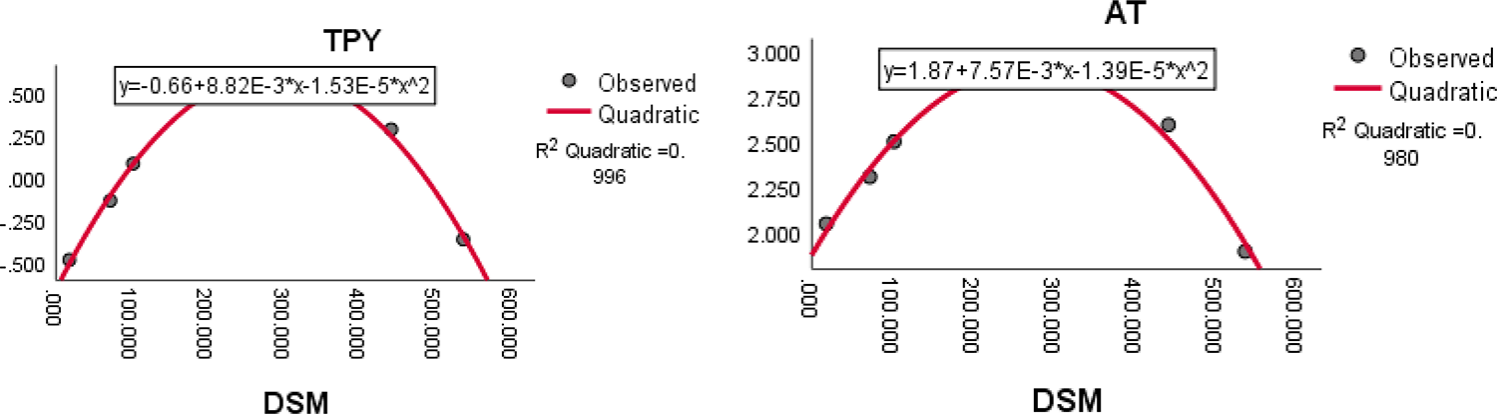
The quadratic regression curve of domination SM index $(DSM)$ against T. Pyroformis $(TPY)$ and oral rat acute toxicity *(AT)*.

The quadratic regression curve plots for the most reliable computed domination index having highest ${R}^{2}$ with $p<0.05$ and ADMET properties of anti-tuberculosis drugs are shown in Figs. 12, 13 and 14.

The observed trend of minimal standard error (from Tables 17 and 20) being significantly lower for ADMET properties compared to physicochemical properties, can be attributed to several factors such as data availability, biological variability, experimental techniques and regulatory requirements. The combination of greater data availability, the influence of biological processes, more precise experimental techniques, and regulatory requirements contribute to the observed trend of lower standard errors for ADMET properties compared to physicochemical properties.

Based on the statistical parameters of highly correlated indices with the ADMET properties and their regression plots, the following results are summarized.

(i) The domination terminal Weiner index (DTW) is most suitable for predicting the intestinal absorption. (IA)
(ii) The domination Schultz index (DSC) is most suitable for predicting the BBB permeability.
(iii) The domination modified Schultz index (DSC*) is most suitable for predicting the maximum tolerated dose (MTD)
(iv) The domination Ashwini index (DA) is most suitable for predicting the water solubility (WS)
(v) The domination SM index (DSM) most suitable for predicting the fraction unbound (FU), oral rat acute toxicity (AT) oral rat chronic toxicity (CT) and T. Pyriformis (TPY).

## Conclusion

The concept of domination is of significant importance when exploring the structural attributes of molecular graphs. A complete understanding of the molecular structures can be attained by analysing their dominations and their topological indices. In this article, eight domination distance-based topological indices are computed for isoniazid, pyrazinamide, ethambutol, ethionamide, linezolid and levofloxacin. These indices are obtained using the minimum dominating distance matrices of the molecular graphs. Subsequently, these topological indices find application in Quantitative Structure–Property Relationship (QSPR) analysis to probe the physicochemical and ADMET properties of drugs used in tuberculosis treatment. The QSPR analysis through the quadratic regression shows that most of the computed domination numbers of the drugs have a strong predictive ability for physicochemical and ADMET properties of the drugs. The QSPR analysis provides a strong correlation between domination distance-based topological indices and the physicochemical properties, such as boiling point, enthalpy of vaporization, flash point, molar refraction, polarizability, and molar volume. Similarly, the ADMET properties, including water solubility, total clearance, intestinal absorption, and oral rat acute toxicity, T. Pyriformis, CNS permeability, BBB permeability also have high correlation with certain domination distance—based topological indices. These predictions present valuable insight into the molecular characteristics and contribute to strategic decision-making in drug discovery and associated domains.

## Future work

The domination distance-based indices of molecular graphs can be computed to other chemical molecules and drugs used to treat other diseases like cancer, rheumatoid arthritis, diabetes etc., to explore/analyze the properties of the drugs. The analysis of Quantitative Structure–Activity Relationship (QSAR), Quantitative Structure–Reactivity Relationship (QSRR) and Quantitative Structure–Toxicity Relationship (QSTR) can be conducted for various molecular structures utilizing this domination distance-based topological indices. The domination distance-based topological indices could be applied to diverse range of structures, including dendrimers, nanomaterials, polymers, social networks, and biological networks, to explore their chemical properties.

## Data Availability

The datasets generated and/or analysed during the current study are available in the ChemSpider and pkCSM repositories, whose URL are https://www.chemspider.com/ and https://biosig.lab.uq.edu.au/pkcsm/prediction respectively. The chemical structures of the drugs are obtained from National Centre for Biotechnology Information (NCBI) whose URL is https://pubchem.ncbi.nlm.nih.gov/ The Statistical Package for the Social Sciences (SPSS) software package has been used in the research and whose URL is https://www.ibm.com/spss.
